# Biomimetic TME‐Responsive Nanotheranostics for Precise NIR‐II Ratiometric Photoacoustic Imaging and Synergistic Immuno‐Photothermal Therapy

**DOI:** 10.1002/smll.74044

**Published:** 2026-06-02

**Authors:** Xin Li, Hongrui Qiu, Lik Hang Hugo Tse, Xuehan Wang, Hsuan Lo, Huili Wang, Qi Li, Shiying Li, Yanjuan Gu, Wing‐tak Wong

**Affiliations:** ^1^ Department of Applied Biology and Chemical Technology The Hong Kong Polytechnic University Hung Hom Hong Kong China; ^2^ Guangdong Provincial People's Hospital, Guangdong Academy of Medical Science Southern Medical University Guangzhou China; ^3^ The Hong Kong Polytechnic University Shenzhen Research Institute Shenzhen Guangdong China

**Keywords:** IDO inhibition, nanotheranostics, photoacoustic imaging, second near‐infrared (NIR‐II) window, synergistic immuno‐photothermal therapy

## Abstract

The complex immunosuppressive microenvironment of triple‐negative breast cancer (TNBC) remains a major obstacle in attaining long‐term clinical success and halting metastatic progression. To address this, the rational design of tumor microenvironment (TME)‐responsive nanotheranostics is crucial for converting ‘cold’ tumors into ‘hot’ ones through synchronized imaging and precision therapy. Herein, we present a biomimetic, stimuli‐activatable assembly (Au/Ag@HMON‐NLG@CCM) utilizing a H_2_O_2_‐responsive Au/Ag core to facilitate NIR‐II ratiometric photoacoustic (PA) detection and thermal ablation. By encapsulating the IDO inhibitor NLG919 within a hollow mesoporous organosilica (HMON) shell and coating with cancer cell membrane (CCM), the system achieves superior tumor‐targeting and synergistic immuno‐photothermal effects. Specifically, TME‐overexpressed H_2_O_2_‐induces the oxidative degradation of the Ag, shifting the absorption into the NIR‐II window to enable robust ratiometric PA visualization and efficient PTT (η = 42.8%). The resulting hyperthermia triggers immunogenic cell death (ICD) to initiate antigen release, while NLG919‐mediated IDO1 blockade alleviates local immunosuppression, collectively remodeling the ‘cold’ TME. In bilateral tumor models, this integrated strategy effectively suppresses both primary lesions and distant metastases, demonstrating a potent systemic abscopal effect and durable antitumor immunity. Overall, this multifunctional nanoplatform provides a transformative strategy for NIR‐II‐guided precision medicine, offering a robust approach to eradicating metastatic and immunosuppressive malignancies.

## Graphical abstract

A biomimetic Au/Ag@HMON‐NLG@CCM nanotheranostic is engineered for tumor microenvironment‐responsive NIR‐II ratiometric photoacoustic imaging and immune‐photothermal therapy. The integration of hyperthermia‐triggered immunogenic cell death with IDO‐blockade‐mediated metabolic remodeling promotes dendritic cell maturation and T‐cell activation. This strategy elicits potent systemic immunity against primary tumors and distant metastases, demonstrating a robust abscopal effect.

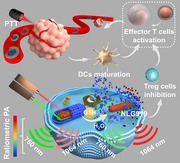

## 1 Introduction

Cancer immunotherapy has become a cornerstone of modern oncology by mobilizing the endogenous immune system to recognize and clear malignant cells and to establish durable immune surveillance against recurrence and metastasis [1]. Despite major clinical successes, a substantial fraction of patients responds poorly because many solid tumors exhibit an immunologically “cold” phenotype, characterized by insufficient antigen presentation and limited infiltration of effector T cells [2]. To elevate immunogenicity of tumor, synergistic integration immunotherapy with antitumor approaches that can promote immunogenic cell death (ICD) in tumors, especially with chemotherapy [3, 4, 5], photodynamic therapy [6, 7], and photothermal therapy (PTT) [8, 9, 10, 11, 12], have been extensively studied. Among the therapeutic modalities, the combination of noninvasive spatially and temporally controlled mild PTT (42°C–49°C) and immunotherapy is preferred for clinical applications as the traditional high‐temperature PTT would cause irreversible non‐specific damage to nearby healthy tissues and strongly upregulate heat shock proteins (HSPs) resulting thermotolerance and limiting antitumor therapeutic efficacy [13]. Nevertheless, the antitumor performance of mild PTT remains constrained by limited light penetration, particularly in larger or deeper tumors, which can result in insufficient heating, incomplete tumor eradication, and subsequent relapse. To address this problem, deeper tissue penetration and enhanced photothermal efficiency can be achieved as the development of applications in the second near‐infrared (NIR‐II, 1000–1700 nm) region. In addition to physical limitations, mild PTT can also trigger compensatory immunoregulatory pathways within tumors, including upregulation of indoleamine 2,3‐dioxygenase (IDO) and PD‐L1, which function as adaptive resistance mechanisms [8, 13]. IDO catalyzes the conversion of tryptophan (Trp) into kynurenine (Kyn), thereby reshaping local metabolism and driving immunosuppression through multiple routes, such as dampening effector T‐cell and natural killer cell function while facilitating the expansion and activation of regulatory T cells (Tregs). The resulting enrichment of highly suppressive Tregs in the tumor microenvironment (TME) can directly limit the cytotoxic activity of CD8^+^ cytotoxic T lymphocytes (CTLs). Accordingly, integrating mild PTT with IDO inhibition is expected to mitigate Treg‐mediated immunosuppression, promote CTL infiltration, and ultimately enhance antitumor immune efficacy.

Benefiting from its deep tissue penetration, high temporal resolution, and high in vivo spatial resolution, precise photoacoustic (PA) imaging‐guided therapy has drawn tremendous attention. Photoacoustic computed tomography (PACT) is one of three major implements, which can be implemented for both microscopic and macroscopic imaging [14]. With introducing externally contrast agent, PACT can visualize targeted biomarkers with high selectivity and sensitivity, making it a valuable platform for monitoring the immune status in TME [5, 15]. Among these, NIR‐II active materials have been extensively employed in PA imaging owing to their reduced photon scattering, minimized background absorption, and enhanced tissue penetration depth [16, 17, 18]. In particular, TME‐activated nanotherapeutics operating in the NIR‐II window have rapidly emerged as a research hotspot in optical imaging [19, 20, 21, 22, 23, 24].

Multifunctional nanotheranostic platforms, integrating diagnostic imaging and therapeutic intervention within a single nanoplatform, have drawn extensive attention in cancer nanomedicine. Nevertheless, most existing nanotheranostic platforms are always in “on” mode, causing severe side effects due to off‐target activation, including relatively low imaging sensitivity and drug delivery efficiency. To overcome these challenges, recognizing the unique biochemical signatures of specific disease sites offers a pathway for stimulus‐responsive activation. Aberrant tumor metabolism creates distinctive microenvironmental features, such as acidity, elevated reactive oxygen species (ROS), and dysregulated redox buffering (e.g., altered glutathione levels), which can serve as endogenous stimuli to trigger on‐demand drug release and enhance imaging signals for better cancer theragnostic [25, 26]. In the landscape of nanomedicine, hollow mesoporous organosilica nanoparticles (HMON) have gained significant recognition for image‐guided oncology due to their exceptional biocompatibility and inherent biodegradability [27, 28, 29]. Current research indicates that the majority of TME‐responsive systems are designed with a single activatable purpose [30, 31], which greatly compromises the accuracy of tumor diagnosis and treatment. Therefore, the rational design and fabrication of TME‐responsive nanotheranostics capable of simultaneous imaging and therapy is of importance for achieving precise tumor diagnostics and effective therapeutic outcomes.

**SCHEME 1:**
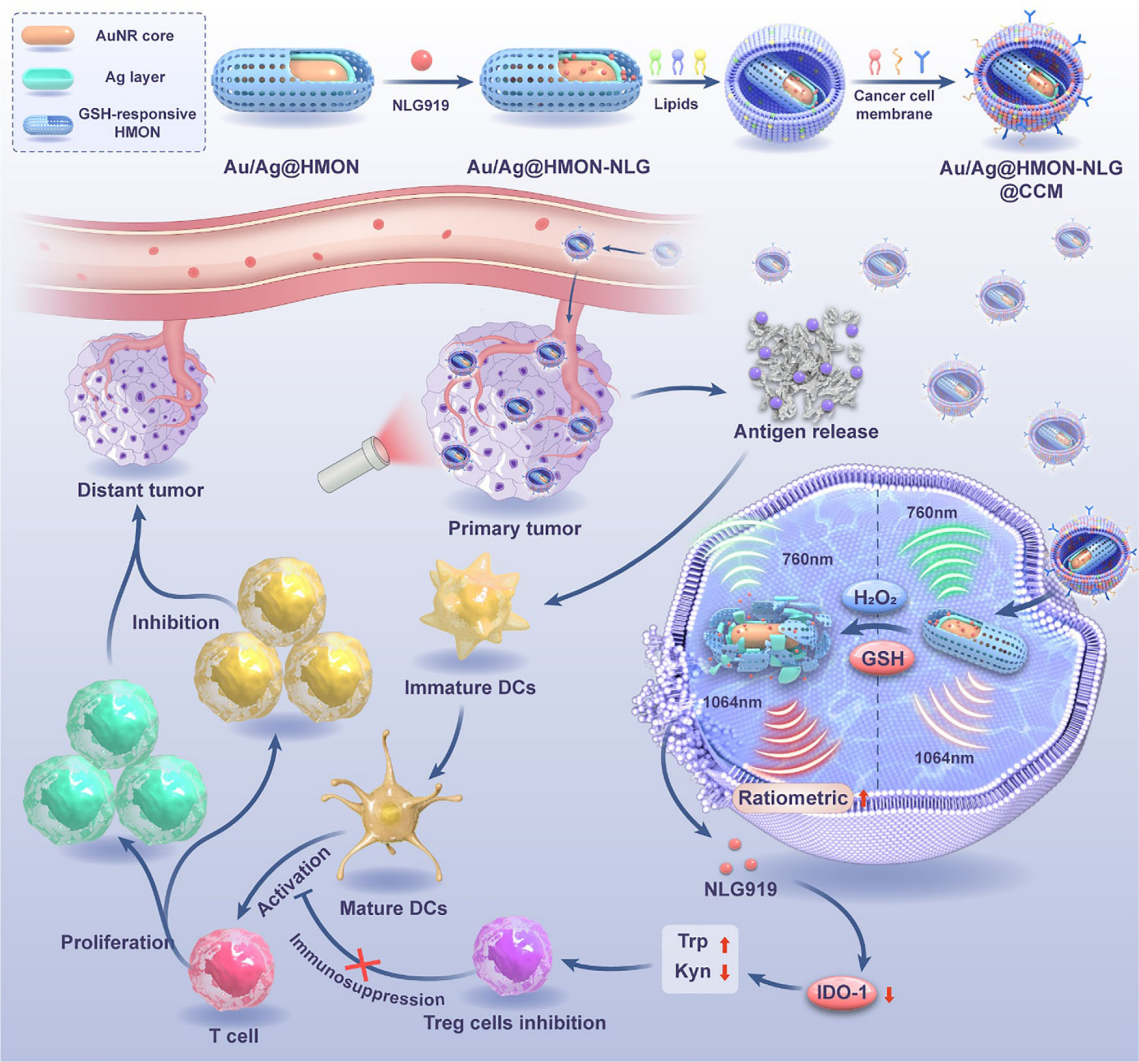
Schematic illustration of preparation process of biomimetic nanotheranostics (Au/Ag@HMON‐NLG@CCM) and its mechanism of action in TME‐responsive NIR‐II ratiometric photoacoustic imaging and synergistic immuno‐photothermal therapy.

Therefore, we report here a sophisticated NIR‐II activatable plasmonic assembly utilizing a silver‐layered gold nanorod encapsulated within an HMON framework (Au/Ag@HMON) that enables simultaneous TME‐responsive NIR‐II ratiometric PA imaging and controlled immunodrug release (Scheme 1). Following the encapsulation of the IDO inhibitor NLG919, a multifunctional nanocomposite (Au/Ag@HMON‐NLG@CCM) was engineered with a self‐assembled lipid (LP) coating and subsequently fused with 4T1 cancer cell membrane (CCM), serving as a gatekeeper to prevent premature release, enhancing physiological stability, and providing therapeutic selectivity. Upon intravenous injection, the Au/Ag@HMON‐NLG@CCM rapidly accumulated in the tumor region due to excellent homologous targeting ability obtained from the 4T1 CCM, and after that, the nanotheranostics achieved a NIR‐II PA “turn on” process due to the oxidation of Ag shell induced by overexpressed H_2_O_2_ in the TME. During this process, the accumulation of Au/Ag@HMON‐NLG@CCM caused the PA signal at 760 and 1064 nm increasing initially, and then the oxidation of Ag shell in Au/Ag@HMON‐NLG@CCM shift the absorption of the system into the NIR‐II window, thereby inducing a decrease in the PA_760_ signal and an increase in the PA_1064_ signal. Given the observed opposite evolution trends in H_2_O_2_‐responsive PA_760_ and PA_1064_ signals, the ratiometric index (PA_1064_/ PA_760_) provides a robust strategy for accurately monitoring the activation state of the material and validating the trigger effect, thereby guiding and evaluating the performance of synergistic photothermal‐immunotherapy. Furthermore, the restoring PTT effect induces immunogenic tumor cell death. Additionally, hyperemia induced by recovered PTT effect, together with overexpressed‐glutathione (GSH), accelerate HMON degradation and enables on‐demand NLG919 release from the nanocomposite, thereby significantly promoting the immunotherapy efficacy.

## 2 Result and Discussion

### 2.1 Design, Synthesis, and Characterization of Nanotheranostics

**FIGURE 1:**
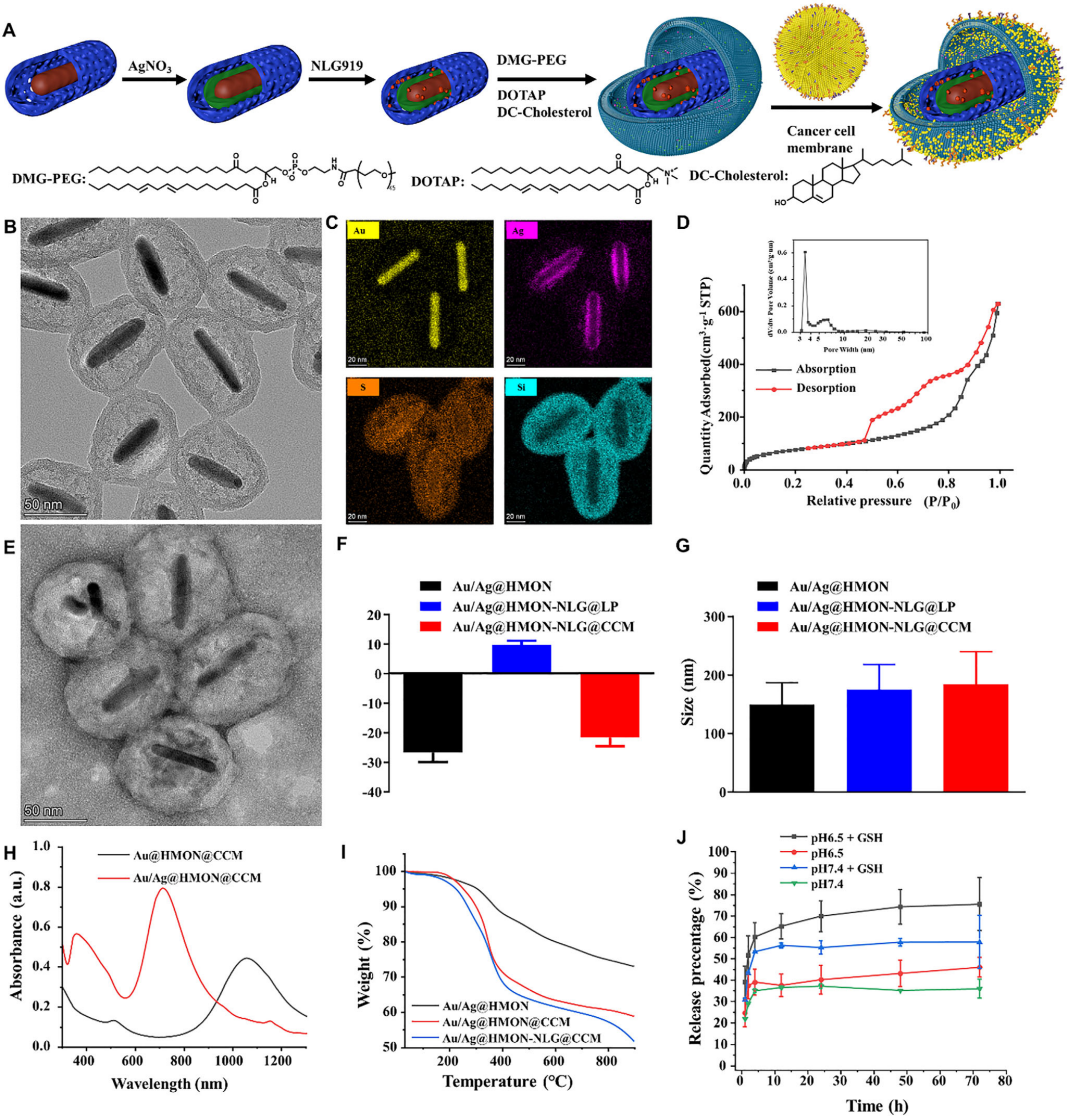
Synthesis and characterization of nanotheranostics. (A) Scheme for the preparation of Au/Ag@HMON‐NLG@CCM. (B) TEM image and (C) EDX mapping of Au/Ag@HMON. (D) N_2_ adsorption‐desorption isotherms of Au/Ag@HMON (inset: pore distribution). (E) TEM image of Au/Ag@HMON‐NLG@CCM. (F) zeta potential and (G) DLS of Au/Ag@HMON, Au/Ag@HMON@LP and Au/Ag@HMON@CCM. (H) UV‐vis‐NIR absorption spectra of Au/Ag@HMON@CCM and Au@HMON@CCM. (I) TGA curves of Au/Ag@HMON, Au/Ag@HMON@CCM and Au/Ag@HMON‐NLG@CCM. (J) Drug releasing profiles in PBS (10 mMm, pH7.4 or 6.5) with or without GSH (10 mm) (n = 3).

The TME‐responsive nanocomposite Au/Ag@HMON‐NLG@CCM was synthesized as schematically outlined in Scheme 1A. The Au@HMON was first prepared according to our previous publication [32]. A thin Ag shell was homogenously deposited onto the surface of gold nanorods (AuNRs) to generate Au/Ag@HMON by reducing Ag^+^ with ascorbic acid under alkaline conditions. Moreover, the thickness of the Ag shell and the position of the surface plasmon resonance (SPR) peak could be tuned by varying the amount of AgNO_3_ added (Figure S1). The Ag layer around Au was confirmed by transmission electron microscope (TEM) due to its lower electron density compared with Au (Figure 1B). In the images, the bright rod‐shape regions were surrounded by duller shells, indicating that the AuNRs core was wrapped with an Ag shell. Interestingly, in our developed method, the formation of Ag nanoparticles on the surface or within the pores of the HMON was completely inhibited when the Ag^+^ and ascorbic acid were added gradually, in contrast to previous report [33]. When the AuNRs surface was coated with an Ag shell of 8.1 nm thickness (width increase from 8.3 ± 1.5 nm to 16.4 ± 2.7 nm and a length increase from 53.7 ± 11.7 nm to 56.7 ± 12.3 nm), the SPR absorption of resulting nanocomposites exhibited a blue‐shift from 1060 to 700 nm. (Figure S1 and Table S1). The presence of Au, Ag, Si and O within the nanocomposites can be confirmed furtherly by EDX elemental mapping of Au/Ag@HMON (Figure 1C), in which the signal of Au cores (yellow) was homogeneously surrounded by the signal of Ag shell (purple). Quantitative analysis based on EDX analysis and ICP‐MS revealed that the mass ratio of Au: Ag was about 1: 0.95, indicating the controlling coating process of Ag layer. Using the N_2_ adsorption‐desorption isotherm method, the BET surface area of Au/Ag@HMON was determined as 281.6 m^2^/g, and the pore size of Au/Ag@HMON was 3.4 nm (Figure 1D). A lipid mixture comprising DMG‐PEG, cholesterol and DOTAP at a mass ratio of 40: 2: 1 was employed to modify HMON to prevent the leakage of NLG919. This was followed by fusion with the 4T1 CCM, which enhanced stability, compatibility and targeting capability of the system. TEM images revealed that Au/Ag@HMON‐NLG@CCM showed increased dimensions with a length of 101.2 ± 16.9 nm and width of 81.2 ± 12.5 nm (Figure 1E; Table S1). The successive surface functionalization of nanocomposites with liposome and CCM was monitored by measuring hydrodynamic size and zeta potential (Figure 1F,G). The hydrodynamic size exhibited stepwise increases from 150.1 ± 36.9 nm (Au/Ag@HMON) to 175.1 ± 43.2 nm after liposome coating (Au/Ag@HMON‐NLG@LP), and further to 183.9 ± 56.5 nm after CCM coating (Au/Ag@HMON‐NLG@CCM). Meanwhile, the zeta potential measurements revealed that the surface charge transitions from −24.4 mV (Au/Ag@HMON) to +11.7 mV (Au/Ag@HMON‐NLG@LP), and subsequently to −17.8 mV (Au/Ag@HMON‐NLG@CCM). This charge reversal phenomenon confirms successful biomimetic coating through the cationic lipid component (DOTAP with quaternary ammonium groups) in the liposome layer imparting positive surface charge, coupled with subsequent anionic biopolymer from the cell membrane restoring negative surface potential. Au/Ag@HMON‐NLG@CCM and Au@HMON‐NLG@CCM exhibited characteristic peaks at 700 and 1060 nm, respectively, indicating that the SPR properties of the nanocomposites were maintained during liposome and CCM modifications (Figure 1H). To determine the percentage of different components in the Au/Ag@HMON‐NLG@CCM, TGA experiments were conducted. The results indicate that the residual weight percentages for Au/Ag@HMON, Au/Ag@HMON@CCM and Au/Ag@HMON‐NLG@CCM are 73.0%, 58.8%, and 52.5%, respectively (Figure 1I; Table S2), and it could be calculated that the mass percentage of Au/Ag@HMON, liposome and NLG919 were 71.7%, 17.3% and 11.0%, respectively. We speculate that the high drug loading capacity may mainly arise from the following factors. First, NLG919 contains hydroxyl group, which readily form hydrogen‐bonding interactions with silanol groups (─SiOH) on the silica surface. Furthermore, NLG919 is a weakly basic drug featuring an imidazole ring connecting to electron‐donor moiety. The imidazole group is more easily protonated in acidic or neutral aqueous solution, resulting in stronger electrostatic attraction with the negatively charged silica surface [34, 35].

An efficient controlled‐release property is among the most critical factors for an ideal carrier. Our pervious study demonstrated the GSH‐ and pH‐responsiveness of HMON [32], and based on these findings, similar conditions were applied in the present study. The NLG919 release kinetics were systematically investigated under physiologically relevant conditions using PBS buffers (pH 7.4 and 6.5) at 37°C, with 10 mm GSH supplementation in designated groups, where the pH 7.4 was simulating normal tissue conditions versus pH 6.5 with 10 mm GSH simulating tumor microenvironments and intracellular matrix (Figure 1J). It revealed that approximately 36% and 38% of the drug was released at pH 7.4 and pH6.5 after 12 h. This release is primarily attributed to NLG919 molecules physically adsorbed onto the external surface and within the shallow mesopores of the HMON framework. In the presence of GSH, release increased to 65% after 12 h, while approximately 24% of NLG919 remained in the delivery system even after 72 h at pH 6.5. The enhanced trigger effect is hypothesized to arise from the biodegradation of HMONs, wherein the tetra‐sulfur linker undergoes cleavage under GSH‐overexpressed and acidic conditions characteristic of the TME, thereby enabling controlled release of the payload specifically at the tumor site. This selective responsiveness not only enhances therapeutic precision but also minimizes off‐target effects.

To examine the stability of the nanoplatform, the size of Au/Ag@HMON@CCM dispersed in PBS and FBS during 7 days of incubation was characterized by DLS (Figure S2A). Although the hydrodynamic diameter rose from 175.1 to 239.6 nm in the PBS group, the nanoplatform remained highly stable size in serum‐containing media, indicating its potential for systemic circulation. The morphology of the nanoplatform after one week of incubation in PBS was examined TEM (Figure S2B), which demonstrated that the CCM coating preserves the structural integrity of the Au/Ag core.

### 2.2 Activatable NIR‐II Photothermal and PA Performance of Au/Ag@HMON@CCM

**FIGURE 2:**
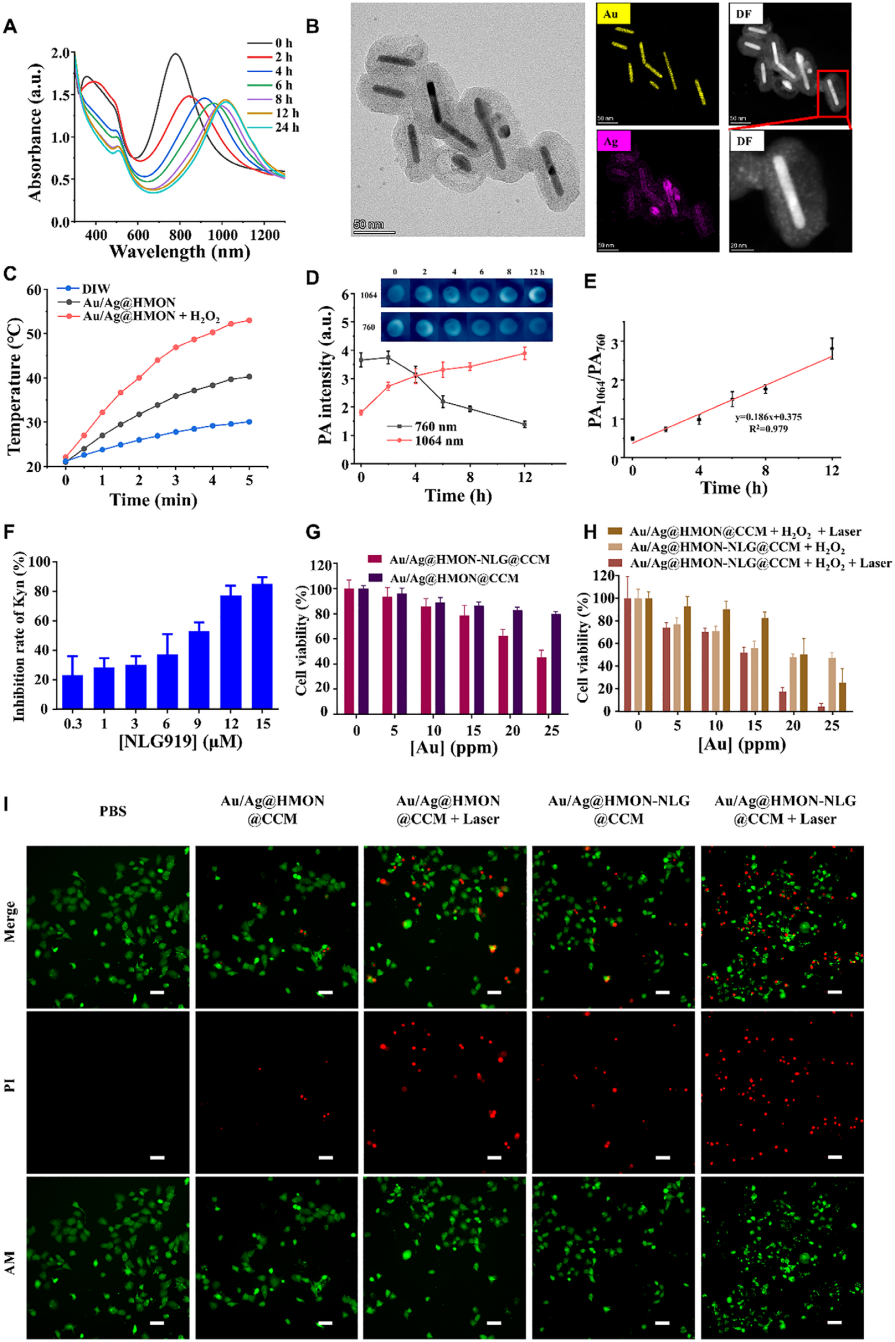
Activatable NIR‐II photothermal and PA properties of Au/Ag@HMON@CCM. (A) UV‐vis‐NIR absorption spectra of Au/Ag@HMON treated with 0.1 mm H_2_O_2_ for different time. (B) TEM image of Au/Ag@HMON treated with H_2_O_2_ for 4 h and corresponding EDX mapping. (C) Temperature profiles of Au/Ag@HMON and Au/Ag@HMON upon NIR‐II laser irradiation after incubation with H_2_O_2_ for 8 h (0.75 W/cm^2^, 5 min) (D, E) The change of PA amplitudes and PA images (D), and the PA_1064_/PA_760_ ratio (E) of the phantom filled with Au/Ag@HMON@CCM (50 ppm Au) in the presence of H_2_O_2_ over time. Data are defined as mean S.D. (n = 3). (F) IDO inhibition in the 4T1 cells with Au/Ag@HMON‐NLG@CCM containing different concentrations of NLG919 treatment (n = 3). (G) Viability of 4T1 cells after incubated with Au/Ag@HMON@CCM and Au/Ag@HMON‐NLG@CCM for 24 h (n = 5). (H) In vitro anticancer activity tests of Au/Ag@HMON@CCM after various treatments (H_2_O_2_: 0.1 mm; 0.75 W/cm^2^, 5 min) (n = 5). (J) Live/dead analysis of 4T1 cells stained by calcein‐AM (green) and PI (red) after different treatments (scale bar: 100 µm).

The triggered responsiveness of Au/Ag@HMON to H_2_O_2_ allowed recovery of intense NIR‐II extinction, enabling a range of valuable functions for the biomedical field. To evaluate the feasibility of Au/Ag@HMON applied as a NIR‐II PA probe, the etching behavior of the Ag shell in the Au/Ag@HMON was evaluated by monitoring the absorption shift via UV‐Vis‐NIR spectrum and quantifying residual content of Ag via ICP‐MS as well (Figure S3). In the presence of 0.1 mm H_2_O_2_ at 37°C, and the SPR extinction peak of Au/Ag@HMON red‐shifted to 1060 nm from 700 nm within 12 h, while the mass ratio of Ag to Au decreased from 0.94 to 0.17. To further confirm the dissolution of the Ag shell inside the nanocomposite, TEM and EDX were performed on samples after incubation with H_2_O_2_. As illustrated in Figure 2B, the Ag shell on the surfaces of the AuNRs was gradually etched upon H_2_O_2_ treatment. Compared with Figure 1C, the silver signal, initially localized around the AuNRs, decreased and redistributed across the hollow mesoporous framework, with some silver even detectable on the outer surface of the HMON. The etching process was further confirmed by dark‐field TEM imaging, where the widespread bright spots indicated crystalline fragments of decomposed silver layer dispersed around the amorphous HMON. The results suggest that H_2_O_2_‐mediated etching of the Ag shell efficiently enhances the NIR‐II optical activity of Au/Ag@HMON, highlighting its potential for responsive imaging applications.

Subsequently, we investigated and evaluated the activation‐dependent enhancement in NIR‐II photothermal conversion and PA signal generation of Au/Ag@HMON following incubation with H_2_O_2_, while the untreated Au/Ag@HMON severed as a contrast. Upon irradiation with a 1064 nm laser (0.75 W cm^−2^, 5 min), the temperature of Au/Ag@HMON solution showed only modest increases (from 22.0°C to 40°C), while the temperature of the H_2_O_2_‐treated Au/Ag@HMON rapidly increased to 55°C (Figure 2C), demonstrating enhanced conversion efficiency following Ag shell removal. The photothermal conversion efficiency of the H_2_O_2_‐treated Au/Ag@HMON nanocomposite was determined to 42.8% using standard calculation method (Figure S4) and negligible signal decay was observed after five on/off laser cycles, indicating robust photostability of the nanocomposites.

The PA performance of Au/Ag@HMON@CCM and Au@HMON@CCM was evaluated using PACT phantom experiments, establishing a linear correlation between nanocomposite concentration and PA intensities at 1064 nm (PA_1064_) and 760 nm (PA_760_) (Figure S5). Moreover, the slope for Au/Ag@HMON@CCM and Au@HMON@CCM at 1064 nm are 0.0235 and 0.0482, respectively, indicating a ∼2‐fold increase in NIR‐II PA signal upon complete oxidation of Ag shell. In contrast, the slopes at 760 nm are 0.0301 and 0.0132, respectively, reflecting a marked reduction. This opposite trend makes the ratio of PA intensities at 1064 to 760 nm (PA_1064_/PA_760_) a reliable ratiometric parameter for monitoring material transitions and validating the trigger effect.

To investigate the response sensitivity of nanoplatform toward H_2_O_2_ levels, PA signal response across 1–1000 µm H_2_O_2_ was evaluated by incubating Au/Ag@HMON@CCM (50 ppm) under these conditions (Figure S6). No obvious PA signal change can be observed at 1 µm of H_2_O_2_. When the concentration reached 10 µm, the PA signal exhibited an obvious increase at 1064 nm accompany by a decrease at 760 nm. With further increases in H_2_O_2_ concentration, the PA signal at 1064 nm progressively intensified and approached its maximum value at 1000 µm. Overall, a clear concentration‐dependent enhancement in PA signal was demonstrated, confirming the responsiveness of the nanoprobe to the oxidative tumor‐like environment.

The ability of Au/Ag@HMON@CCM to trigger TME‐activated NIR‐II PA signals was evaluated by incubating the sample ([Au] = 50 ppm) with 0.1 mm H_2_O_2_. The PA images and corresponding PA intensities of phantom composed of Au/Ag@HMON@CCM + H_2_O_2_ at 1064 and 760 nm with different incubation time were obtained (Figure 2D,E). The PA signals exhibited an increasing trend at 1064 nm and concomitant decreasing trend at 760 nm. This opposing pattern can be attributed to the gradual and efficient oxidation of Ag to Ag^+^ mediated by H_2_O_2_. In vitro ratiometric PA intensities (PA_1064_/PA_760_) were conducted to quantify the responsiveness of the PA signal to H_2_O_2_‐triggered activation. A robust linear correlation (R^2^ = 0.979) was established between the PA_1064_/PA_760_ and the incubation time, suggesting the potential of this system for real‐time guidance in optimizing the timing of in vivo synergistic therapeutic interventions. The H_2_O_2_‐activated enhancements in photothermal conversion efficiency and PA signals of Au/Ag@HMON@CCM demonstrated that the developed nanocomposites could act as promising TME‐triggered NIR‐II nanotheranostic agents, effectively integrating PTT with PA imaging.

### 2.3 In Vitro Biocompatibility and IDO Inhibition and Synergistic Immuno‐Photothermal Therapy Evaluation

To characterize the inhibition of the IDO activity by the developed nanotheranostics at the cellular level, the concentrations of Kyn in the supernatant medium of 4T1 cells were determined following treatment with varying concentrations of Au/Ag@HMON‐NLG@CCM (Figure 2F). The inhibition rate exhibited a concentration‐dependent increase, reaching approximately 84.8% upon treatment with 15 µm of NLG919. These findings suggest that NLG919 can be effectively released from nanocomposites within tumor cells, thereby alleviating antitumor immunosuppression and enabling synergistic PTT and immunotherapy.

Before validating the application of Au/Ag@HMON‐NLG@CCM with laser irradiation in vitro, the cytotoxicity of Au/Ag@HMON@CCM was first investigated by MTT method. The Au/Ag@HMON@CCM has a negligible effect on the viability of 4T1 cells, fibroblast 3T3 cells and macrophage RAW264.7 cells after 24 h of incubation at concentrations upon to 25 ppm (Figure 2G; Figure S7), confirming the good biocompatibility. In contrast, the presence of NLG919 (Au/Ag@HMON‐NLG@CCM) induced a concentration‐dependent cytotoxic response. Furthermore, the therapeutic effect of Au/Ag@HMON‐NLG@CCM was evaluated under conditions supplemented with exogenous H_2_O_2_ (Figure 2H). Compared to the Au/Ag@HMON‐NLG@CCM + H_2_O_2_ or Au/Ag@HMON@CCM + H_2_O_2_ + Laser groups, the Au/Ag@HMON‐NLG@CCM + H_2_O_2_ + Laser group displayed a significantly reduced cell viability, demonstrating a superior synergistic therapeutic effect arising from hyperthermia induced by restored NIR‐II laser irradiation in combination with immunotherapy triggered by the released NLG919. To visually demonstrate the synergistic therapeutic efficiency, cells from different samples (PBS, Au/Ag@HMON@CCM and Au/Ag@HMON‐NLG@CCM) were treated with or without laser irradiation, followed by staining of live cells with green‐emissive calcein‐AM and dead cells with red‐emissive propidium iodide (PI), respectively (Figure 2J). In the Au/Ag@HMON‐NLG@CCM + H_2_O_2_ + Laser group, green fluorescence reduced significantly while red fluorescence was enhanced significantly, indicating that the combination of immunotherapy with PTT effectively induce apoptosis in H_2_O_2_‐overexpressed TME.

### 2.4 In Vivo Fluorescence and PACT Imaging

Accurate tumor localization and the identification optimal treatment windows play a critical role in maximizing the efficacy of antitumor therapy. The tumor targeting behavior of Au/Ag@HMON‐ICG@CCM was first assessed through in vivo fluorescence imaging. The biodistribution of ICG‐labelled Au/Ag@HMON‐ICG@CCM and Au/Ag@HMON‐ICG@LP was intravenously injected for in vivo fluorescence imaging (Figure S8). The fluorescence intensity of the tumor site in Au/Ag@HMON‐ICG@CCM‐treated mice was stronger than that in Au/Ag@HMON‐ICG@LP‐treated mice during the monitored time range, demonstrating efficient tumor‐targeting capability. Moreover, Au/Ag@HMON‐ICG@CCM exhibited peak tumor accumulation within the tumor at 6 h post‐injection, followed by predominant hepatic clearance. The CCM‐mediated endocytosis possibly contributes substantially to the tumor‐specific accumulation. This selective accumulation at targeted sites would effectively minimize off‐target distribution, thereby reducing adverse effects on normal cells and tissues.

**FIGURE 3:**
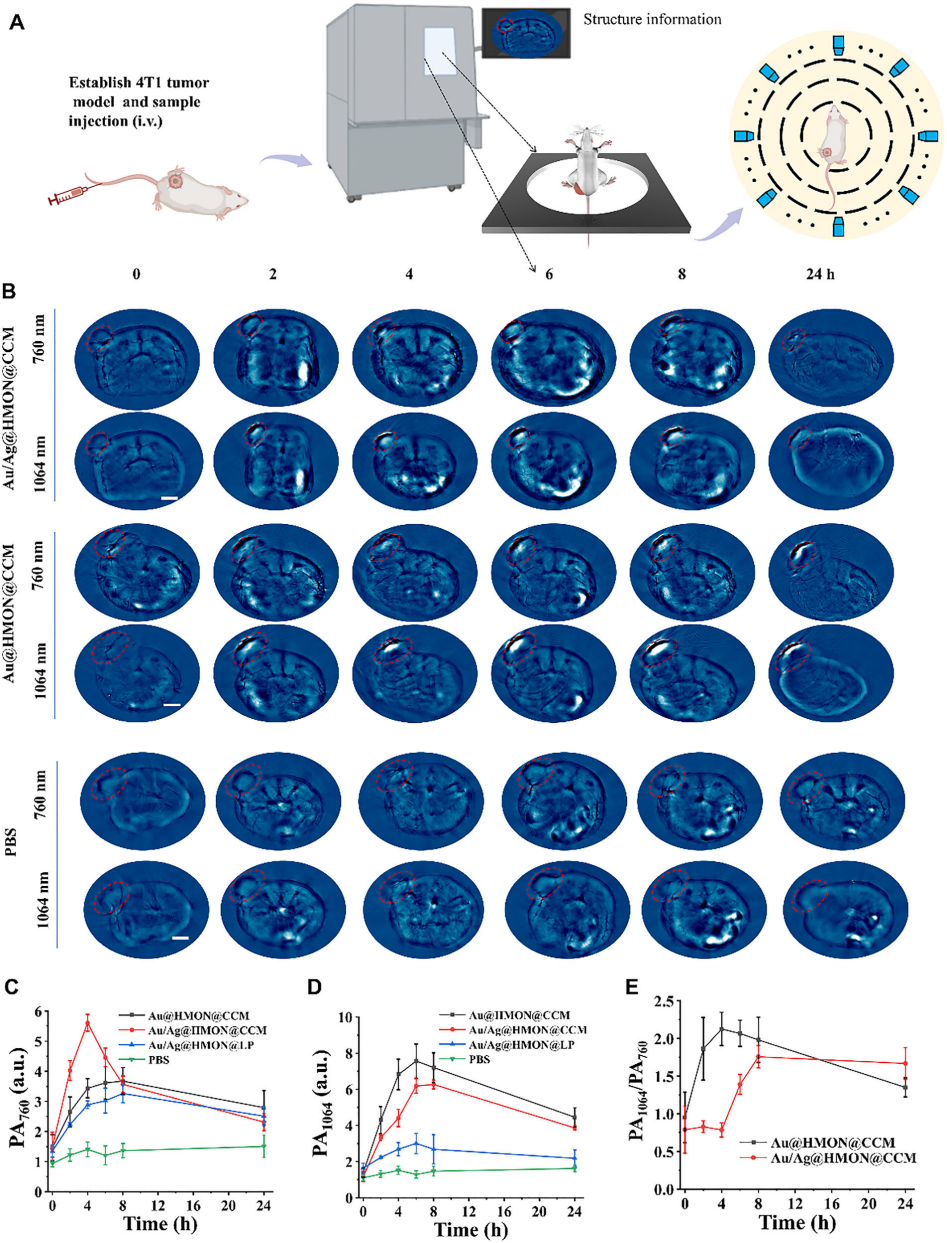
In vivo PACT images of 4T1 tumor‐bearing mice before and after intravenous injection of Au/Ag@HMON@LP, Au@HMON@CCM and Au/Ag@HMON@CCM. (A) Scheme of PACT imaging system. (B) Representative PACT images at 760 and 1064 nm at different post‐injection time. (C,D) Quantification of the PA intensities at (C) 760 nm and (D) 1064 nm in the tumor region as a function of post‐injection time of nanoprobes. (E) The ratiometric PA signals (PA_1064_/PA_760_) with the post‐injection time. Data are defined as mean S.D. (n = 3).

In vivo PACT imaging was also employed to monitor and assess the capacity of H_2_O_2_‐activated NIR‐II PA imaging for nanocomposites (Figure 3; Figure S9). Mice received intravenous administration of Au/Ag@HMON@CCM, with Au/Ag@HMON@LP and Au@HMON@CCM as controls. The time‐dependent evolution of PA signals at 760 and 1064 nm from tumor sites were then collected (Figure 3B–D). The tumor‐site accumulation rate, as indicated by PA_760_, was much lower in the Au/Ag@HMON@LP compared to the Au/Ag@HMON@CCM, thereby demonstrating the targeting capability mediated by the CCM (Figure 3C). Moreover, in the Au@HMON@CCM group, the PA_760_ signal increased gradually over time, reaching a plateau at 8 h. Since the PA_760_ signal for this nanocomposite is insensitive to H_2_O_2_, the observed increase in PA_760_ signal should be attributed to enhanced absorption at 760 nm resulting from the efficient tumor accumulation of Au@HMON@CCM. In contrast, the enhanced PA_760_ signal could be initially obtained in the tumor site within 4 h after injection of Au/Ag@HMON@CCM, with a subsequent gradual decline, which can be ascribed to the synergistic effect of the continuous accumulation of Au/Ag@HMON@CCM at the tumor region and oxidation of Ag into Ag^+^ ions. Moreover, a similar changing trend was observed in the PA_1064_ signal for Au@HMON@CCM and Au/Ag@HMON@CCM, except for the difference in PA_1064_ intensity (Figure 3D). They both showed that PA_1064_ increased gradually over time, reached a plateau at 6–8 h, and subsequently decreased gradually. To identify optimal time for 1064 nm laser treatment, we compared in vivo ratiometric PA intensities (PA_1064_/PA_760_) of Au@HMON@CCM and Au/Ag@HMON@CCM. As shown in Figure 3E (red curve), PA_1064_/PA_760_ ratio exhibits no obvious change at 4 h post‐injection, then begins to increases as Ag oxidation progresses, and by 8 h the ratio becomes comparable to that of Au@HMON@CCM, suggesting that Ag oxidation is essentially complete. The different evolutionary trend of PA_1064_/PA_760_ over post‐injection time for two nanoprobes may provide guidance for selecting 8 h post‐injection as the suitable time points for laser irradiation (Figure 3E). These specific ratiometric PA imaging responses allow for precise tumor “localization‐timing” to support highly efficient imaging‐guided immuno‐photothermal therapy.

### 2.5 PTT/IDO‐Activity Inhibition Suppress the Primary Tumor and Distal Tumor In Vivo

**FIGURE 4:**
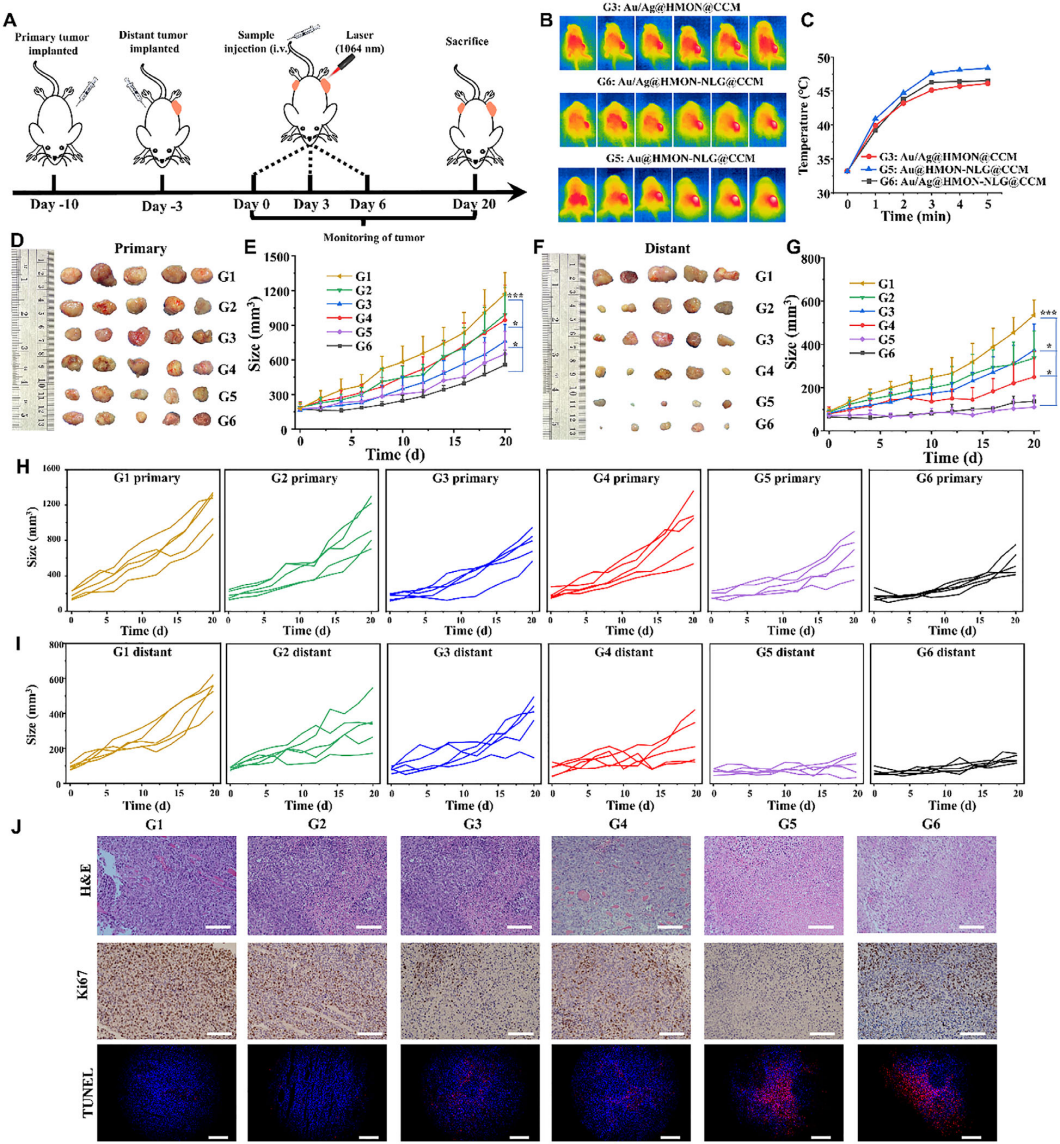
In vivo Au/Ag@HMON‐NLG@CCM‐mediated PTT and immunotherapy on bilaterally 4T1 tumor‐bearing mice. (A) Scheme of constructing bilateral tumor model and treatment procedure for PTT and immunotherapy. (B,C) Thermal images and corresponding temperature profiles of tumor sites after i.v. injection of nanotheranostics. (D,E) Representative images and average volume change curves of primary tumors after different treatments. (F,G) Representative images and average volume change curves of distant tumors after different treatments. (H,I) Volume change curves of primary (H) and distant tumors (I) in each mouse after different treatments (Groups: G1‐PBS, G2‐Au/Ag@HMON@CCM, G3‐Au/Ag@HMON@CCM + laser, G4‐Au/Ag@HMON‐NLG@CCM, G5‐Au@HMON‐NLG@CCM + laser, G6‐Au/Ag@HMON‐NLG@CCM + laser). (J) H&E, ki67 and TUNEL staining images of distant tumor tissues acquired at 21 days. The scale bars are 100 µm. The data are presented as mean ± SD; ^*^ *p* < 0.05, ^**^ *p* < 0.01, ^***^ *p* < 0.001, ^***^ *p* < 0.0001; n = 5.

The in vivo synergistic therapeutic effect of Au/Ag@HMON‐NLG@CCM was first investigated using bilaterally 4T1 tumor‐bearing BALB/c mice while the primary and distant tumors were constructed on day ‐10 and ‐3. The model establishment procedures and subsequent treatment are summarized in Figure 4A. These mice were divided to 6 groups randomly: G1‐PBS, G2‐Au/Ag@HMON@CCM, G3‐Au/Ag@HMON@CCM + Laser, G4‐Au/Ag@HMON‐NLG@CCM, G5‐Au@HMON‐NLG@CCM + Laser, G6‐Au/Ag@HMON‐NLG@CCM + Laser. All groups underwent tail vein injections on days 0, 3, and 6 post‐tumor implantations respectively, with the identical concentration (Au: 3.1 mg kg^−1^; NLG919: 2.8 mg kg^−1^) for each group. For laser‐treated groups (G3, G5, G6), primary tumors were exposed under the 1064 nm laser (1.5 W cm^−2^, 5 min) at 8 h post‐injection. Afterward, the growth curves of the primary tumors and distant tumors were recorded for 3 weeks. Under irradiation with the 1064 nm laser, the surface temperatures in G3, G5 and G6 reached about 46°C–48°C (Figure 4B,C), which demonstrated that these nanotheranostics can be applied to mild PTT. Both primary and distant tumor dimensions were quantitatively monitored every 2 days. For primary tumors (Figure 4D,E), while monotherapy approaches, which include Au/Ag@HMON@CCM‐based PTT (G3) and Au/Ag@HMON‐NLG@CCM‐mediated immunotherapy (G4), demonstrated modest growth retardation compared to PBS control (G1), neither achieved significant tumor suppression. Synergistic effects emerged in the combinatorial group (G6), where concurrent immunotherapy and PTT yielded significant tumor suppression. Additionally, comparative evaluation between G6 (Ag‐containing) and G5 (Ag‐free) showed no significant difference in primary tumor volumes, suggesting limited contribution of silver ions to therapeutic outcomes under these conditions.

For distant tumors (Figure 4F,G), therapeutic responses exhibited distinct patterns: PTT alone (G3) showed negligible anti‐tumor activity, while immunotherapy monotherapy (G4) induced modest but non‐significant growth delay, demonstrating the contribution of immune adjuvants in driving antitumor immunity. Moreover, only combinational groups (G5/G6) demonstrated robust abscopal effects, achieving comparable distant tumor inhibition rates, with minimal regrowth observed. The robust abscopal effect against distant tumors is driven by systemic CTLs overcoming the less‐fortified barriers of distant sites, following the relief of primary immunosuppression. Furthermore, while G5 and G6 exhibit comparable therapeutic efficacy, the integration of Ag in G6 is mechanistically essential for optimizing NIR‐II photoacoustic imaging, thereby realizing a high‐resolution theranostic platform without compromising biosafety. Notably, the body weights of mice showed no significant variations throughout the treatment period (Figure S10). Furthermore, hematoxylin and eosin (H&E) and Ki67 staining were performed on tumor sections to investigate the antitumor effect in depth (Figure 4J; Figure S11), revealing that G6 treatments could cause severe tumor tissue damage and inhibit tumor proliferation. Consistent with these findings, terminal deoxynucleotidyl transferase‐mediated dUTP nick‐end labeling (TUNEL) analysis confirmed extensive programmed cell death within the experimental cohorts compared to the control group, highlighting the therapeutic advantage of the G6 protocol. To evaluate systemic safety, H&E staining was performed on vital organs including the heart, liver, spleen, lungs, and kidneys, which revealed no discernible structural damage and verified the high biocompatibility of the nanotheranostics (Figure S12). The long‐term efficacy was further supported by Kaplan‐Meier survival curves, which showed that mice in the G5 and G6 groups achieved significantly higher survival rates than those in the other treatment categories throughout the study (Figure S13). To further evaluate the residual metal levels in vivo, the biodistribution of Ag and Au in major organs was quantified by ICP‐MS (Figure S14), while the metal contents had returned to baseline levels at day 7, indicating that it had been effectively cleared from the body and thus would not cause Ag‐related toxicity. Finally, a broad assessment of biochemical parameters confirmed the absence of acute toxicity, with liver function markers (AST and ALT) and kidney/systemic health indicators (UREA, CREA, UA, and CK) all remaining within normal ranges following various therapeutic interventions (Figure S15). No obvious abnormalities were observed, indicating that nanotheranostics of Au/Ag@HMON‐NLG@CCM combined with laser has no obvious toxicity to liver, kidney and other organs, confirming its high biosafety and low‐risk characteristics for effective cancer treatment.

### 2.6 T‐Cell Mediated Antitumor Immune Activation and Immunosuppression Remodeling

**FIGURE 5:**
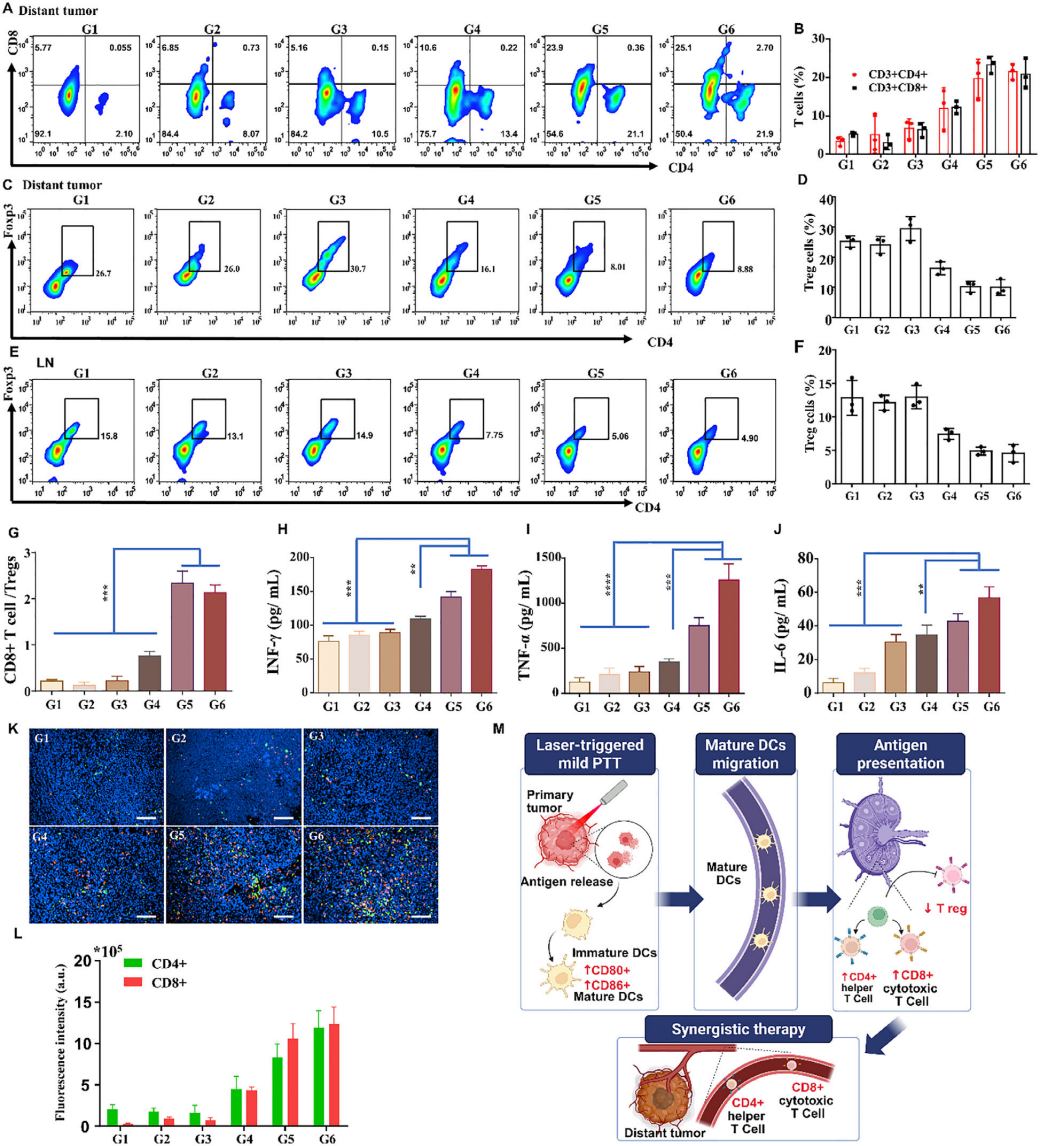
In vivo antitumor immune response evaluated by flow cytometry and cytokine analysis. (A,B) Representative flow cytometry plots and quantitative analysis of CD4^+^ and CD8^+^ tumor‐infiltrating T cells within the gated CD3^+^ population (CD3^+^CD4^+^ and CD3^+^CD8^+^) in distant tumor tissues after various treatments. (C,D) Representative flow cytometry plots and quantitative analysis of CD3^+^CD4^+^Foxp3^+^ regulatory T (Treg) cells in distant tumors after different treatments. (E–G) Representative flow cytometry plots (E), quantitative analysis (F) of CD3^+^CD4^+^Foxp3^+^ Treg cells and (G) ratio of Treg CD8^+^ T cells in lymph nodes (n = 3). (H–J) Levels of immune activation‐related cytokines IFN‐γ (H), TNF‐α (I), and IL‐6 (J) in distant tumor tissues following different treatments (n = 3). (K,L) Representative immunofluorescence staining images and statistical analysis of CD4 (green) and CD8 (red) in distant tumor sections from different treatment groups, demonstrating T‐cell infiltration and immune activation. (M) Mechanism of therapy pathway illustrating key biomarker in flow cytometry. (^*^ *p* < 0.05, ^**^ *p* < 0.01, ^***^ *p* < 0.001, ^***^ *p* < 0.0001).

To further elucidate the adaptive immune responses induced by different treatments, T‐cell infiltration and cytokine secretion in distant tumors and lymphoid organs were systematically analyzed by flow cytometry, immunofluorescence staining, and ELISA (Figure 5). As shown in Figure 5A,B, the proportion of cytotoxic T lymphocytes (CTLs, CD3^+^CD8^+^) in distant tumors was significantly increased after treatment with NLG919‐based immunotherapy. In particular, the single immunotherapy group (G4) exhibited a marked elevation in CTL infiltration (12.2%) compared with the PBS group (G1, 5.3%). Notably, the highest CTL levels were observed in the combinational therapy groups G5 (23.3%) and G6 (20.9%), suggesting a synergistic enhancement of T‐cell‐mediated antitumor immunity attributable to the combined effects of IDO1 inhibition induced by NLG919 and PTT. Meanwhile, helper T cells (CD3^+^CD4^+^) were also significantly increased in the G5 (21.1%) and G6 (21.9%) groups compared to G1(2.1%), indicating the function of helper T cell enhanced significantly, achieving the activation and proliferation of CTLs while promoting anti‐tumor immunity.

Given that CD4^+^ T cells comprise both effector and immunosuppressive regulatory subsets, the frequencies of regulatory T cells (Tregs, CD3^+^CD4^+^Foxp3^+^) were further examined to dissect this duality. Flow cytometry analysis of distant tumors (Figure 5C,D) and lymph nodes (Figure 5E,F) revealed a pronounced reduction in Treg populations in the combinational therapy groups. Specifically, the percentages of Tregs in lymph nodes and distant tumors decreased to 4.9% and 10.1% in G5, and further to 4.5% and 9.9% in G6, respectively, compared with substantially higher levels in PBS group (G1, 12.8% in lymph nodes and 25.2% in distant tumors). An intermediate decrease was observed in the G4 group (7.4% in lymph nodes and 16.3% in distant tumors), indicating that immunotherapy alone partially alleviated immunosuppression. In contrast, the PTT‐only group showed a slight increase in Treg frequency, consistent with previous reports that photothermal‐induced inflammation may recruit regulatory immune components. To comprehensively evaluate the balance between effector and suppressive immune responses, the ratios of CD8^+^ T cells to Tregs in distant tumors were quantitatively analyzed (Figure 5G). The highest CD8^+^/Treg ratios were observed in the G5 (2.8) and G6 (2.6) groups, confirming a favorable shift toward antitumor immune activation and suggesting that the immunomodulatory effect of NLG919 was further amplified by PTT.

In parallel, the levels of key immune activation‐related cytokines in distant tumor tissues were quantified by ELISA. As shown in Figure 5H–J, the concentrations of IFN‐γ, TNF‐α, and IL‐6 were significantly elevated in the G6 group compared with all other treatment groups. These cytokines are critical mediators of cytotoxic T‐cell function and inflammatory immune responses, indicating that Au/Ag@HMON‐NLG@CCM‐mediated PTT elicited a robust antitumor immune activation. Notably, single‐modality treatments induced only moderate cytokine secretion, further highlighting the synergistic immune‐enhancing effect of the combined therapeutic strategy. Finally, immunofluorescence staining of distant tumor sections corroborated the flow cytometry findings. As shown in Figure 5K,L, tumors from the G5 and G6 groups exhibited dense infiltration of CD4^+^ (green) and CD8^+^ (red) T cells throughout the tumor parenchyma, whereas sparse T‐cell distribution was observed in control and single‐treatment groups. These results provide spatial confirmation of enhanced T‐cell recruitment and activation within the TME. Collectively, these data demonstrate that the combination of PTT and IDO1‐targeted immunotherapy effectively promotes cytotoxic T‐cell infiltration, suppresses regulatory T‐cell‐mediated immunosuppression, and induces a proinflammatory cytokine milieu, thereby establishing a robust systemic antitumor immune response (Figure 5M).

### 2.7 Dendritic Cell Maturation Induced by Synergistic Therapy in Tumors and Lymphoid Organs

**FIGURE 6:**
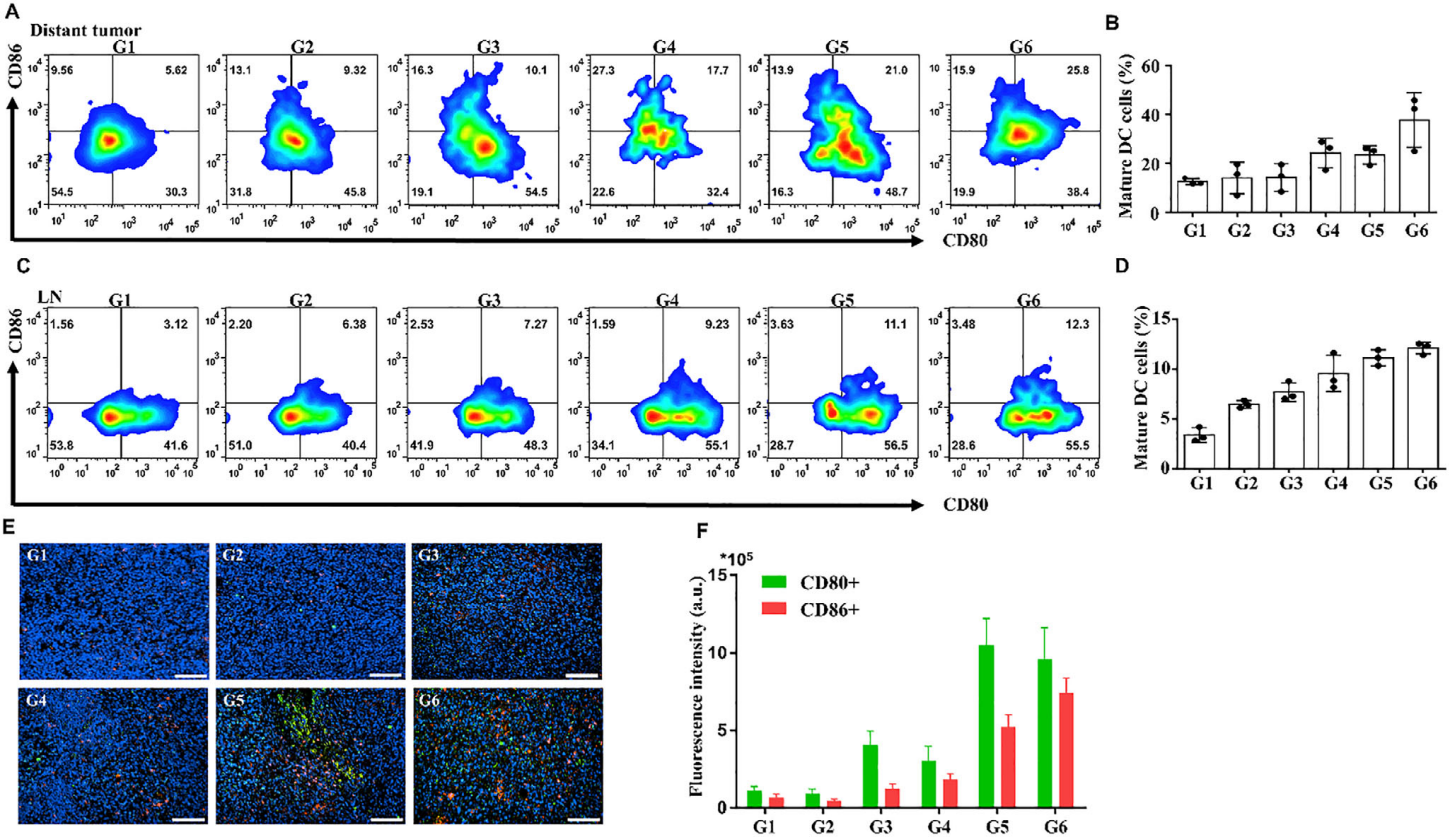
Dendritic cells (DCs) maturation in distant tumors and lymph nodes. (A,B) Representative flow cytometry plots and statistical analysis of CD80^+^ and CD86^+^ mature dendritic cells within the gated CD11c^+^ population (CD11c^+^CD80^+^ and CD11c^+^CD86^+^) in distant tumors. (C,D) Representative flow cytometry plots and statistical analysis of CD80^+^ and CD86^+^ mature DCs within the gated CD11c^+^ population (CD11c^+^CD80^+^ and CD11c^+^CD86^+^) in lymph nodes. (E,F) Representative immunofluorescence staining images statistical analysis of CD80 (green) and CD86 (red) in distant tumor sections from different treatment groups, indicating DCs maturation.

To investigate immune responses initiated by dendritic cell (DC) activation, tumors and lymphoid organs were harvested and analyzed by flow cytometry and immunofluorescence staining (Figure 6; Figure S16). Tumor‐draining lymph nodes were first collected to evaluate DC maturation induced by different treatments. As shown in Figure 6A,B, the proportion of mature DCs (CD11c^+^CD80^+^ and CD11c^+^CD86^+^) in lymph nodes was markedly increased after combination therapy. Notably, the G6 group exhibited the highest DC maturation level, reaching 12.0%, which was approximately 3.6‐fold higher than that of the PBS group (G1, 3.36%). A comparable maturation level was observed in the G5 group (11.1%, 3.3‐fold increase versus G1). These values were significantly higher than those observed in groups receiving partial treatments, including G4 (9.5%, 2.8‐fold), G3 (7.7%, 2.3‐fold), and G2 (6.4%, 1.9‐fold). Consistent maturation trends were further observed in distant tumors (Figure 6C,D), where the G6 group demonstrated a 2.9‐fold increase in mature DCs compared with the PBS group, exceeding those of G3 (1.9‐fold) and G4 (1.1‐fold). Similar enhancements in DC maturation were also detected in primary tumors (Figure S17) and spleen tissues (Figure S18), with the G6 group consistently showing the highest activation level. Notably, as shown in Figure 6A,C, the proportion of CD80 single‐positive cells was significantly higher than that of CD86 single‐positive and double‐positive cells. Immunologically, the higher proportion of CD80 over CD86 single‐positive cells indicates a late‐stage, stable maturation profile, as CD80 is typically upregulated following the early expression of CD86. This prolonged CD80 expression plays a critical role in sustaining durable T‐cell activation, thereby supporting the long‐term suppression of distant tumors. These results collectively indicate that the combination therapy effectively amplifies DC maturation across both local and distant immune compartments, thereby facilitating systemic immune activation.

Immunofluorescence staining of distant tumor sections further corroborated the flow cytometry results (Figure 6E,F). Strong and colocalized expression of CD80 (green) and CD86 (red) was observed in tumors from the G6 group, whereas only weak signals were detected in control and single‐treatment groups. This spatial evidence confirms that DC maturation is maximized under combination therapy, in agreement with the quantitative flow cytometry data. Overall, these findings demonstrate that while single photothermal therapy or immunotherapy alone induces only modest DC maturation, their combination synergistically promotes robust DC activation in tumors and lymphoid organs. Such enhanced dendritic cell maturation provides a critical immunological foundation for subsequent T‐cell priming and systemic antitumor immune responses.

### 2.8 Transcriptomic Analysis of the Antitumor Mechanism

**FIGURE 7:**
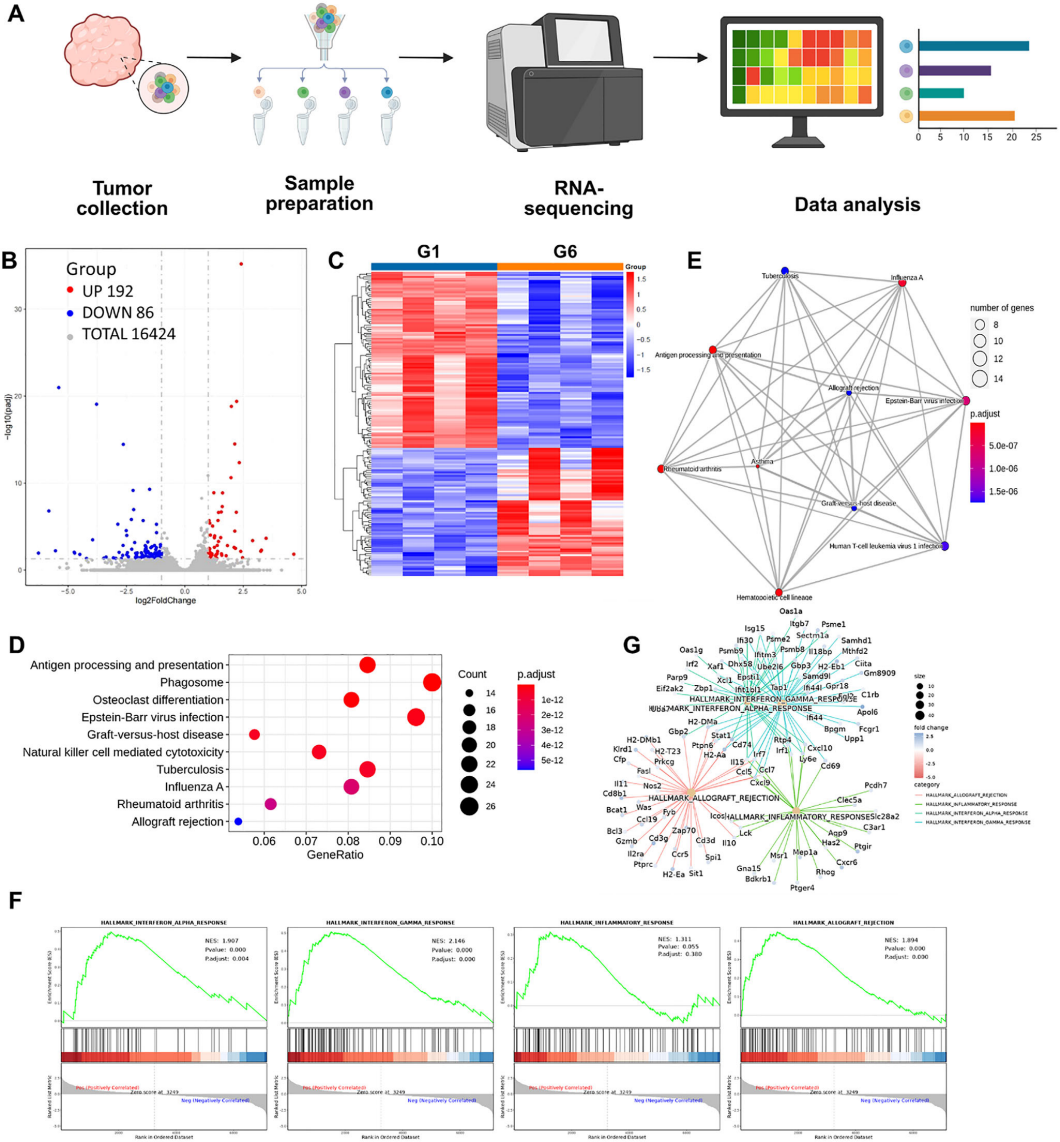
(A) Scheme of transcriptomic analysis of 4T1 tumor tissues after Au/Ag@HMON‐NLG@CCM treatment. (B) Volcano plot illustrating differentially expressed genes (DEGs) between G6 and G1. Gene set enrichment analysis of Hallmark gene sets: (C) Heat map of relative gene expression levels of G6 and Control. (D) KEGG enrichment of varied signaling pathways in G6. (E) KEGG pathway enrichment network analysis of genes in G6 compared to the control group. (F) Representative pathways enriched in the specific gene in G6 as GSEA database. (G) Enrichment map in G6 as GSEA database.

To further explore the mechanism of antitumor immune induced by the combination of PTT and immunotherapy, distant tumor tissues were harvested for RNA‐sequencing transcriptomic analyses. The volcano plot illustrates the number of differentially expressed genes (DEGs) of G6 compared with the control group, 278 genes in the G6 exhibited significant expression differences, including 192 upregulated genes and 86 downregulated genes (Figure 7B). The heatmap reveals that unsupervised clustering based on the expression patterns of these top genes clearly segregated the treated tumors from the control group, indicating a distinct transcriptional state induced by the therapy (Figure 7C). Subsequently, Kyoto encyclopedia of genes and genomes (KEGG) enrichment analysis revealed the significant enrichment of three key pathways related to immune (Figure 7D): antigen processing and presentation, which enhances tumor antigen visibility to the adaptive immune system; natural killer cell mediated cytotoxicity, indicating the mobilization of innate immune effector cells; and allograft rejection, a hallmark signature of potent, CTL‐mediated immune killing. Furthermore, an enrichment network visualized the tight functional interconnectivity among these activated pathways, demonstrating a broad and integrated immune activation module (Figure 7E). Figure 7E,F displays the top gene set enrichment analysis (GSEA). The individual GSEA enrichment plots confirm these upregulated gene sets related to immune activation, including interferon‐alpha response (NES = 1.907), interferon‐gamma response (NES = 2.146), inflammatory response (NES = 1.311), and allograft rejection (NES = 1.894), indicating a coherent type I and II interferon‐driven immune response, activating cytotoxic T cell effector functions in the TME (Figure 7F). The enrichment map revealing a highly interconnected network of these immunotherapy‐associated gene sets, and the significant overlap of genes among these hubs indicates they function not in isolation but as an integrated module (Figure 7G). These findings demonstrate that the combination of PTT with immunotherapy provoked a wide‐spread anticancer immune activation that strengthened immunotherapy efficacy, with involvement of T cell‐associated immunity.

## 3 Conclusions

In summary, we developed an intelligent, TME‐responsive nanotheranostics (Au/Ag@HMON‐NLG@CCM) that simultaneously enables enhanced NIR‐II ratiometric PA imaging to guide improved synergistic immuno‐photothermal therapy. In vivo imaging studies indicated that Au/Ag@HMON@CCM could be specially triggered by TME to activate its NIR‐II ratiometric PA imaging performance, which provided guidance for selecting 8 h post‐injection as the suitable time point for PTT‐based combination therapy. Together with GSH‐ and pH‐responsive triggers, the released NLG919 reduces Kyn accumulation and limits the expansion of Tregs induced by ineffective PTT, thereby reversing the immunosuppressive TME. In vitro and in vivo therapeutic data show the Au/Ag@HMON@CCM with laser irradiation has almost no obvious adverse effects on normal tissues and can effectively inhibited tumor proliferation. In summary, the intelligent Au/Ag@HMON‐NLG@CCM allows a very precise diagnosis and effective cancer therapy, offering promising prospects for advancing NIR‐II PTT‐based combination strategies toward clinical application.

## 4 Experimental Section

### 4.1 Materials

NaBH_4_, hexadecyl trimethyl ammonium bromide (CTAB), hexadecyl trimethyl ammonium chloride (CTAC), HAuCl_4_∙3H_2_O, AgNO_3_, hydroquinone, triethanolamine, trichloroacetic acid (TCA) were purchased from Sigma. HNO_3_ (67%) was purchased from Avantor. Ascorbic acid was purchased from Alfa Aesar. Ammonia water (28%), HCl (37%), and NaOH were obtained from Duksan. Diethanolamine, tetraethylorthosilicate (TEOS), bis[3‐(triethoxysilyl)propyl] tetrasulfide (BTES) and Ehrlich's reagent were purchased from Macklin. NLG919, IDO‐1 primary antibody, and β‐actin antibodies were purchased from Abcam. The lipids (include dimyristoyl Glycerol‐Polyethylene Glycol 2000 (DMG‐PEG), 3β[N(N',N'Dimethylaminoethane)carbamoyl]cholesterol (DC‐cholesterol), and 1,2‐Dioleoyl‐3‐trimethylammonium‐propane chloride (DOTAP)) were purchased from Dieckmann. Dulbecco's Modified Eagle's Medium (DMEM), fetal bovine Serum (FBS), penicillin‐streptomycin (PS), 4′,6‐diamidino‐2‐phenylindole (DAPI), calcerin acetoxymethyl ester (calcein‐AM), propidium iodide (PI), and methylthiazolyldiphenyl‐tetrazolium bromide (MTT) were purchased from ThermoFisher Scientific. The antibodies including APC‐anti‐mouse CD3, FITC‐anti‐mouse CD8, PE‐anti‐mouse CD4, BV421‐anti‐mouse Foxp3, FITC‐anti‐mouse CD11c, BV421‐anti‐mouse CD86 and APC‐anti‐mouse CD80 for cytometry were purchased from Biolegend. ELISA kits for IFN‐ɣ TNF‐𝛼, IL‐6 were purchased from Biolegend.

### 4.2 Characterization

To evaluate the morphology, internal structure, and elemental mapping of the synthesized nanoparticles, transmission electron microscopy (TEM) was performed using a JEM‐2100 system and Thermo scientific Talos F200X system. The hydrodynamic particle size and zeta potential of nanoparticles were determined using Malvern Nano ZS. The N_2_ adsorption/desorption isotherms were characterized by Micrometitics Tristar 3000 system. Absorption spectrum in UV–vis–NIR range were acquired on an UV‐3600 spectrophotometer (Shimadzu, Japan). Thermal degradation and mass ratios were assessed using a TGA/DSC 3+ analyzer. Concentrations of Au and Ag were measured by an inductively coupled plasma‐mass spectrometry (ICP‐MS, AGILENT 7900). The fluorescence microscope images were captured with a Nikon AX R microscope, while a Nikon Eclipse Ti2‐E was utilized for live‐cell imaging. A Fluke Ti450 camera was used for thermal monitoring. The absorbances of sample prepared in 96‐well microplate were measured using a CLARIOstar multimode microplate reader (BMG Labtech, Germany). Flow cytometry experiment was conducted in FongCyte^TM^2 flow cytometer (Challenbio, China).

### 4.3 Ethical Statements

The animal experiments conducted in this work were approved by the Animal Subjects Ethics Sub‐committee of The Hong Kong Polytechnic University (Case No. 23–24/656‐ABCT‐R‐NSFC). The procedure is in accordance with the guidelines and regulations of Animal Center of The Hong Kong Polytechnic University.

### 4.4 Synthesis of Au/Ag@HMON

The Au@HMON was prepared as published work in our group [32]. To coat the silver layer onto the surface of the AuNRs, the Au@HMON was diluted by diethanolamine solution (0.25 m) until the concentration of Au was about 0.2 mm and heated at 37°C. Typically, AgNO_3_ (30 µL, 100 mm) was added to 60 mL of Au@HMON, followed by addition of ascorbic acid (30 µL, 20 mm) to reduce Ag (I) to Ag (0) to form Au/Ag@HMON. Repeat the step until the absorption peak of Au/Ag@HMON was blueshift to designed region. The product was centrifuged at 15000 rpm and washed with Milli‐Q water and re‐dispersed in 1.2 mL of Milli‐Q water for further usage.

### 4.5 Synthesis of Au/Ag@HMON‐NLG@CCM

The as‐synthesis Au/Ag@HMON (0.6 mL) was centrifuged and redispersed in EtOH (6 mL), and NLG919 (6 mg) was added and stirred for 24 h. Subsequently, Milli‐Q water (12 mL) was added to above solution, and EtOH (3 mL) containing DMG‐PEG (24 mg), cholesterol (1.2 mg) and DOTAP (0.6 mg), was slowly injected over 30 min. The self‐assembled liposome layer on the surface of Au/Ag@HMON was formed through the gradual removal of ethanol. The product, Au/Ag@HMON‐NLG@LP, purified by centrifugation at 8000 rpm and rinsed with Milli‐Q water to eliminate excess lipid and NLG919, and finally redispersed in PBS (1.5 mL).

The 4T1 cancer cell membrane (CCM) vesicles were prepared according to our previous report [32, 36]. For the CCM coating process, Au/Ag@HMON@LP (0.5 mL) was mixed with the equal volume of prepared CCM vesicles, followed by extrusion 20 times through polycarbonate membrane with 200 nm pores. After removing excess CCM by centrifugation at 8000 rpm, Au/Ag@HMON‐NLG@CCM was redispersed in PBS and stored at 4°C for further usage. For the control groups, Au/Ag@HMON@CCM and Au@HMON‐NLG@CCM were prepared using the similar protocol.

### 4.6 H_2_O_2_‐responsive Performance of Au/Ag@HMON@CCM

To investigate the H_2_O_2_‐responsive performance of Au/Ag@HMON@CCM, it was incubated with 100 µm H_2_O_2_ under stirring at room temperature ([Au] = 20 ppm). Reaction solutions were collected at multiple time intervals (0, 2, 4, 6, 8, 12, and 24 h). To characterize the oxidation process, UV‐vis‐NIR absorption spectroscopy (300–1400 nm wavelength range) and photoacoustic signal were measured.

### 4.7 In Vitro Biocompatibility and Immuno‐Photothermal Therapy Synergistic Effect of Nanotheranostics

Murine cancer cell line 4T1, fibroblast cell line 3T3 and macrophage cell line RAW264.7 were obtained from the American Type Culture Collection (ATCC). The cells were cultured in DMEM supplemented with 10% FBS and 1% PS in an incubator at 37°C and 5% CO_2_ atmosphere. To evaluate the biocompatibility of Au/Ag@HMON@CCM, 3T3 fibroblast and RAW264.7 macrophages were cultured and incubated with the nanoplatform for 24 h, and cytotoxicity was assessed by MTT assay.

To examine the synergistic therapeutic efficacy, 4T1 cells were seeded in a 96‐well plate (2.0 × 10^4^ cells per well) and cultured for 24 h. Subsequently, the medium was replaced by fresh DMEM containing 100 µm H_2_O_2_ and different concentration of Au/Ag@HMON‐NLG@CCM or Au/Ag@HMON@CCM ([Au] = 0, 5, 10, 15, 20, 25 ppm) for 24 h. For the PTT groups, after 6 h of incubation, each well was irradiated under laser (0.75 W cm^−2^ 5 min), followed by continued incubation until 24 h. After treatment, the growth media was replaced by MTT solution for additional 4 h of incubation. The crystalized formazan crystals in each well were dissolved with 150 µL of DMSO. Finally, the absorption at 570 nm was acquired by the CLARIOstar microplate reader.

### 4.8 In Vitro Immuno‐Photothermal Therapy Synergistic Effect of Nanotheranostics

To evaluate Kyn inhibition at the cellular level, 4T1 cells were seeded in a 96‐well plate (5 × 10^3^ cells per well) for overnight. 50 ng/mL IFN‐γ and 100 µm Trp were added as substrate to induce IDO expression for 48 h. The DMEM was removed completely, and fresh DMEM was added and incubated for 24 h, which contained Au/Ag@HMON‐NLG@CCM with different concentration of NLG919 (0 to 15 µm). The supernatants (100 µL) were collected after centrifuge at 2000 g for 10 min, and 30% TCA (50 µL) was added. Kyn was obtained by hydrolyzing N‐formylkynurenine at 50°C for 30 min, followed by collecting the supernatant (100 µL) centrifugation at 6000 g for 15 min. Ehrlich's reagent (100 µL) was added to the supernatants and incubated at ambient condition for 30 min. Microplate reader was used to measure the absorbance at 490 nm to quantify the Kyn concentrations.

### 4.9 Calcein AM/ PI Co‐Staining Assay

4T1 cells were seeded into 12‐well plates for overnight incubation. The DMEM was then replaced with freshly prepared culture medium containing Au/Ag@HMON@CCM or Au/Ag@HMON‐NLG@CCM ([Au] = 6 ppm) and cells were incubated for additional 24 h either in the presence or absence of laser irradiation. For the PTT group, cells were irradiated with the 1064 nm laser (0.75 W cm^−2^, 5 min) following a 6 h treatment. Subsequently, the cells were rinsed and stained with Calcein‐AM (10 mg mL^−1^) and PI (5 mg mL^−1^) for 30 min. The stained cells were imaged using an inverted fluorescence microscope.

### 4.10 In Vitro and In Vivo PACT Imaging

The in vitro and in vivo PACT imaging experiments were achieved through a SIP‐PACT system, and 1064 and 760 nm were selected as characteristic wavelength. For in vitro PACT imaging, phantom samples containing Au/Ag@HMON@CCM ([Au] = 0, 25, 50, 100, 200 ppm) were prepared for PACT measurement to establish the quantitative correlation between PA signal and Au concentration. Subsequently, Au/Ag@HMON@CCM treated with H_2_O_2_ for different incubation time (0, 2, 4, 6, 8, and 24 h) were prepared for PACT measurements to quantify the time‐dependent evolution of PA signal. Furthermore, Au/Ag@HMON@CCM ([Au] = 50 ppm) was incubated with different concentrations of H_2_O_2_ (1, 10, 100, and 1000 µm) for 8 h and quantify the PA intensities at 760 and 1064 nm to evaluate the responsive threshold.

For the in vivo PACT imaging, Au/Ag@HMON@CCM and Au@HMON@CCM (100 µL, [Au] = 300 ppm) were injected into tumor‐bearing mice (n = 3). PA signals at the tumor site were monitored before and different post‐injection time points with 20 scanning frames per mm. Throughout the imaging experiment, the mice were anesthetized with 2% isoflurane and kept at 37°C. More details of the PACT system, imaging and reconstruction parameters were in the supporting information.

### 4.11 Antitumor Efficacy Analysis

The antitumor effect was evaluated by bilateral 4T1‐bearing model: primary tumors were constructed by injecting 4T1 cells (1 × 10^6^) into the right backs of female mice aged 6 weeks at day‐10, and distant tumors were constructed at day ‐3 in left backs with identical condition. At day 0, mice (primary tumor volumes ∼150 mm^3^) were randomly divided into six groups: G1‐PBS, G2‐Au/Ag@HMON@CCM, G3‐Au/Ag@HMON@CCM+laser, G4‐Au/Ag@HMON‐NLG@CCM, G5‐Au@HMON‐NLG@CCM+laser, G6‐Au/Ag@HMON‐NLG@CCM+laser. Mice received intravenous administration of samples (NLG919: 2.8 mg kg^−1^) via the tail vein at intervals of 3 days for three injections. In the PTT groups (G3, G5, and G6), tumors were under exposure of the 1064 nm laser (1.0 W cm^−^^2^, 5 min) at 8 h post‐administration, while temperature in tumor region was monitored in real‐time using an infrared thermal camera. The survival curve was obtained by measuring and recording the tumor volumes and the body weights of mice every two days until 35 days. To obtain the biosafety information, after 21 days of treatment, the mice were euthanized and blood, tumor and main organs (heart, liver, spleen, lung and kidney) were harvested. All of the above‐mentioned tissue samples were fixed for Hematoxylin and eosin (H&E), TdT‐mediated dUTP nickend labeling (TUNEL) assay, Ki67 staining and immunofluorescence staining.

Additionally, we prepared 4‐µm‐thick paraffin‐embedded tumor sections from mice in different groups. The sections were subjected to baking, dewaxing, antigen retrieval, and blocking of endogenous peroxidases and nonspecific binding proteins. Subsequently, to indicate the DC maturation, the slices were incubated for 1 h with primary antibodies CD80 and CD86; on the other hand, to indicate the T cell distribution, other slices were incubated with primary antibodies CD4 and CD8. Then, they were incubated with fluorescent‐labeled secondary antibodies (AlexaFluor‐488, AlexaFluor‐594) after rinsing with PBS. After staining nuclei with DAPI, fluorescence images were captured with a 40x oil immersion objective. To access in vivo DC cell maturation and cytokine levels, the mice of different group were sacrificed and tumors together with tumor‐draining lymph nodes were subsequently harvested. The single‐cell suspensions were obtained by mechanically dissociation, and filtration through a nylon mesh (200‐mesh), and incubated with APC‐anti‐mouse CD3, FITC‐anti‐mouse CD8, PE‐anti‐mouse CD4, BV421‐anti‐mouse Foxp3, and APC‐anti‐mouse CD80 and FITC‐anti‐mouse CD11c, BV421‐anti‐mouse CD86, for flow cytometric analysis.

### 4.12 ELISA Analysis for Cytokine Evaluation

After euthanizing the mice following 14 days of treatment across various groups, tumors were excised and immediately weighed. Tumor tissues were then homogenized in PBS according to the volume recommended by the ELISA kit's instructions, using a tissue homogenizer (Bertin, France). The supernatants after centrifugation (12 000 g, 20 min) were collected for evaluation of cytokines. ELISA kits were used to quantify the concentrations of cytokines, including IL‐6, TNF‐α and IFN‐γ, in the tumor microenvironment, according to the instructions from manufacturer. All procedures were performed under standardized conditions to ensure consistency and accuracy.

### 4.13 Transcriptomic MRNA Sequencing

4T1 tumor tissues collected 21 days after treatment with Au/Ag@HMON‐NLG@CCM (NLG919: 2.8 mg kg^−1^; Au: 3.1 mg kg^−1^) were subjected to high‐throughput RNA sequencing to analyze gene expression difference.

### 4.14 Statistical Analysis

Data was presented are expressed as mean ± SD from 5 independent trials, with 3 replicates. Statistical analyses were processed by GraphPad Prism 9.3.0 software (GraphPad Software Inc.) and Flow Jo 10.0 (Flow Jo Software Inc.). Significance analysis between groups were processed by ANOVA or Student's t‐tests, and denoted as ^*^ *p* < 0.05, ^**^ *p* < 0.01, ^***^ *p* < 0.001, ^****^ *p* < 0.0001, indicating a significant difference between groups.

## Conflicts of Interest

The authors declare no conflicts of interest.

## Supporting information




**Supporting File**: smll74044‐sup‐0001‐SuppMat.docx.

## Data Availability

The data that supports the findings of this study are available in the supplementary material of this article.
