# Supplementary material for: Biomimetic TME‐Responsive Nanotheranostics for Precise NIR‐II Ratiometric Photoacoustic Imaging and Synergistic Immuno‐Photothermal Therapy
Source: Small. 2026 Jun 2;22(41):e74044. doi: 10.1002/smll.74044 (PMC13392700; doi:10.1002/smll.74044)
Supplement: Supplementary file 1 — Supporting File: smll74044‐sup‐0001‐SuppMat.docx. [file SMLL-22-e74044-s001.docx]

**Biomimetic TME-Responsive Nanotheranostics for Precise NIR-II Ratiometric Photoacoustic Imaging and Synergistic Immuno-Photothermal Therapy**

*Xin Li ^1^, Hongrui Qiu ^3^, Lik Hang Hugo Tse ^1^, Xuehan Wang ^1^, Hsuan Lo^1^, Huili Wang ^3^, Qi Li ^3^, Shiying Li ^*2^, Yanjuan Gu^* 1,2^, Wing-tak Wong ^* 1,2^*

^1^ Department of Applied Biology and Chemical Technology, The Hong Kong Polytechnic University, Hung Hom, Hong Kong, China. ^2^ The Hong Kong Polytechnic University Shenzhen Research Institute, Shenzhen, 518057, Guangdong, China. ^3^ Guangdong Provincial People's Hospital, Guangdong Academy of Medical Science, Southern Medical University, Guangzhou, 510091, China.

**Correspondence**: Shiying Li (5811sl@gmail.com), Yanjuan Gu (Yanjuan.gu@polyu.edu.hk), Wing-tak Wong([w.t.wong@polyu.edu.hk](mailto:w.t.wong@polyu.edu.hk)).

1. **Experimental sections**

**Characterization of Au/Ag@HMON and Au/Ag@HMON@CCM by thermogravimetric analysis** (**TGA)**

To determine the mass fraction of the organic component in Au/Ag@HMON and Au/Ag@HMON@CCM, TGA was performed on the freeze-dried samples. Typically, approximately 1 mg of the sample was used to measure weight loss as the temperature increased from 30°C to 800°C at a rate of 10°C/min under a nitrogen atmosphere with a flow rate of 60 mL/min.

**Characterization of drug loading efficiency and *in vitro* pH, GSH and NIR-responsive drug release**

To determine drug loading efficiency, Au/Ag@HMON-NLG@CCM was dissolved in MeOH to release the encapsulated NLG919 and measured quantitatively by mass spectrometry. To determine the drug release kinetics profiles, it’s assessed by dialyzing 1 mL of Au/Ag@HMON-NLG@CCM (MWCO 10 kDa) in 4 mL of PBS containing 50 μL of DMSO (pH 7.4 and 6.5) with or without 10 mM of GSH. For the NIR-responsive group, the dialyzed tubes were exposed to laser with 0.75 W/cm^2^ for 5 minutes after 1 hour. For all the groups, solutions (50 μL) were collected as designated time points and analyzed by mass spectrometry.

**Stability measurement of Au/Ag@HMON@CCM**

To evaluate the stability of Au/Ag@HMON@CCM, it was dispersed in PBS and FBS respectively, and these solutions were measured by dynamic light scattering (DLS) at room temperature for different time point intervals (0, 1, 2, 3, 5 and 7 days).

**Measurement of photothermal performance of Au/Ag@HMON@CCM before and after response to H_2_O_2_**

The NIR-II photothermal characteristics of Au/Ag@HMON after response to H_2_O_2_ were investigated under 1064 nm laser irradiation through two experimental paradigms. Au/Ag@HMON@CCM ([Au]= 30 ppm) was incubated with H_2_O_2_ for 12 h and exposed to 0.75 W/cm² irradiation for 5 min, with real-time thermal profiling performed via infrared thermography at 30-sec intervals. The cooling curve was continuously recorded until ambient temperature to calculate the photothermal conversion efficiency (η). The identical conditions were applied for DIW and Au/Ag@HMON@CCM without incubation with H_2_O_2_ as control groups.

**Animal model and preparation**

Orthotopic xenograft tumor models were established in 4-6-week-old mice through sequential implantation. Primary tumors were constructed via right flank subcutaneous injection of 4T1 cells (2×10⁶ cells suspended in 100 μL PBS). Tumor volume progression was monitored and when the volume of primary reached about 80 mm³, secondary implantation was performed in the left flank with identical cell number and volume. Further experiment was started when the primary tumors reached about 150 mm³.

***In vivo* biodistribution analysis**

After the tumor volume reached approximately 150 mm^3^, the 4T1 tumor-bearing mice were treated with ICG-labelled Au/Ag@HMON-ICG@CCM and Au/Ag@HMON-ICG@LP (3.1 mg kg^-1^ Au) via tail vein injection. Then, fluorescence images were obtained via the PerkinElmer IVIS spectrum at scheduled time intervals (0, 0.5, 1, 2, 4, 6,8, 12, and 24 h) post injection.

**Transcriptomic mRNA sequencing and bioinformatics analysis**

Tumor tissues were collected from mice subjected to various treatments for transcriptomic mRNA sequencing. Sequencing data were processed to generate expression levels represented as fragments per kilobase of exon per million mapped fragments (FPKM). The raw sequencing data underwent cleaning, quality control, and normalization before downstream analysis. Differentially expressed genes (DEGs) were identified using a significance threshold of p < 0.05, with the fold-change cutoff defined as the mean fold change plus two standard deviations, based on absolute fold-change values.

Functional enrichment analyses, including Gene Ontology (GO) terms, Kyoto Encyclopedia of Genes and Genomes (KEGG) pathways, and Gene Set Enrichment Analysis (GSEA), were performed to explore the biological functions and pathways associated with DEGs. GO and KEGG analyses were conducted using the clusterProfiler package in R software. For GSEA, annotated gene sets “c2.all.v7.5.1.symbols.gmt” and “c6.all.v7.5.1.symbols.gmt” were downloaded from the Molecular Signatures Database (MSigDB). To explore protein-protein interactions (PPIs) among DEGs, a PPI network was constructed using data from the STRING database. The network was visualized using Cytoscape software (Version 3.8.2), with key hub genes identified through the cytoHubba algorithm. The expression patterns of specific gene sets were further analyzed and visualized as heatmaps using the pheatmap package in R software (3.8.1).

**Biosafety Test**

Biochemical analysis of serum was processed to evaluate the functionality of various organs of health mice treated with different groups. The liver function was evaluated by analyzing the levels of albumin (ALB), alanine aminotransferase (ALT), aspartate aminotransferase (AST), and alkaline phosphatase (ALP). The renal function was evaluated using blood urea nitrogen (BUN) and creatinine (CREA), and the gallbladder function was evaluated through the measurement of total bilirubin (TBIL). Other main organs were fixed in 4% paraformaldehyde, and then H&E stain was used to observe the pathological changes.

1. **Tables**

Table S1. Lengths and widths of Au@HMON, Au/Ag@HMON and Au/Ag@HMON@CCM measured by TEM when 0.1 mM AgNO_3_ used.

| Samples | Au | Au/Ag | Au/Ag@HMON | Au/Ag@HMON@CCM |
| --- | --- | --- | --- | --- |
| Length (nm) | 53.7 ± 11.7 | 56.7 ± 12.3 | 87.8 ± 7.4 | 101.2 ± 16.9 |
| Width (nm) | 8.3 ± 1.5 | 16.4 ± 2.7 | 64.5 ± 4.0 | 81.2 ± 12.5 |

Table S2. Percentage of residual weight of Au/Ag@HMON-NLG@CCM based on TGA curves shown in Figure 1I.

| Samples | Au/Ag@HMON | Au/Ag@HMON@CCM | Au/Ag@HMON-NLG@CCM |
| --- | --- | --- | --- |
| Residual mass percentage (%) | 73.0% | 58.8% | 52.5% |

As the TGA curve of Au/Ag@HMON, Au/Ag@HMON@CCM and Au/Ag@HMON-NLG@CCM, the residual mass ratios are 73.0%,58.8% and 52.5% respectively. The residual component should only be metal (Au and Ag) and inorganic SiO_2_ in both sample and other organic components are removed, i.e., the residuals in all measurements are same so we could obtain the following equation:

$$\frac{\frac{m_{Au/Ag+SiO2}}{m_{Au/Ag@HMON}}}{\frac{m_{Au/Ag+SiO2}}{m_{Au/Ag@HMON@CCM}}}=\frac{73.0\%}{58.8\%}$$

$$\frac{m_{Au/Ag@HMON@CCM}}{m_{Au/Ag@HMON}}= \frac{73.0\%}{58.8\%} \Rightarrow\frac{m_{Au/Ag@HMON}+m_{CCM}}{m_{Au/Ag@HMON}}= \frac{73.0\%}{58.8\%} \Rightarrow m_{Au/Ag@HMON} : m_{CCM}=1:0.24$$

$$\frac{\frac{m_{Au/Ag+SiO2}}{m_{Au/Ag@HMON}}}{\frac{m_{Au/Ag+SiO2}}{m_{Au/Ag@HMON-NLG@CCM}}}=\frac{73.0\%}{52.5\%}$$

$$\frac{m_{Au/Ag@HMON@CCM}}{m_{Au/Ag@HMON}}= \frac{73.0\%}{58.8\%} \Rightarrow\frac{m_{Au/Ag@HMON}+m_{CCM}}{m_{Au/Ag@HMON}}= \frac{73.0\%}{52.5\%} \Rightarrow m_{Au/Ag@HMON} : m_{CCM+NLG}=1:0.39$$

$$\Rightarrow m_{Au/Ag@HMON} : m_{CCM} : m_{NLG}=1 : 0.24 : 0.15=71.9\% :17.3\% : 10.8\%.$$

1. **Figures**


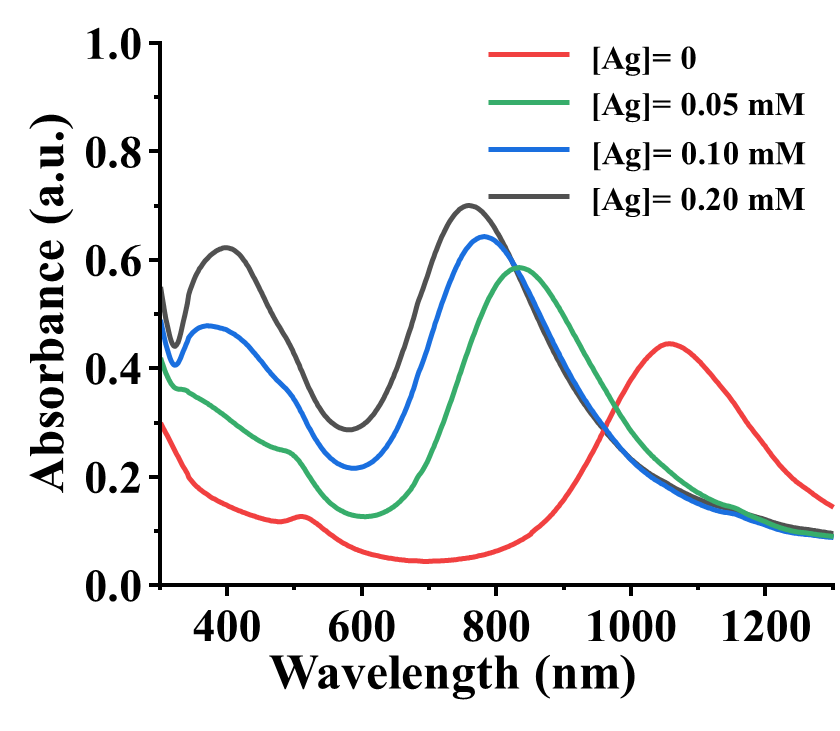


Figure S1. UV-VIS-NIR absorption of Au/Ag@HMON with addition of different amount of AgNO_3_.


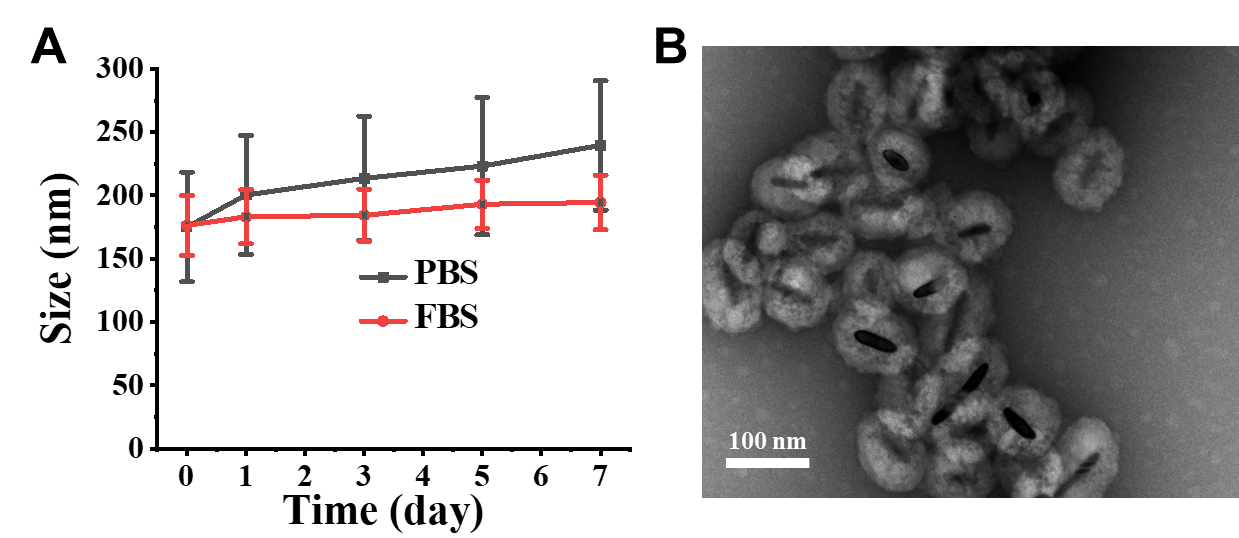


Figure S2. (A) DLS and (B) TEM image of Au/Ag@HMON-NLG@CCM dispersed in PBS after 7 days incubation.


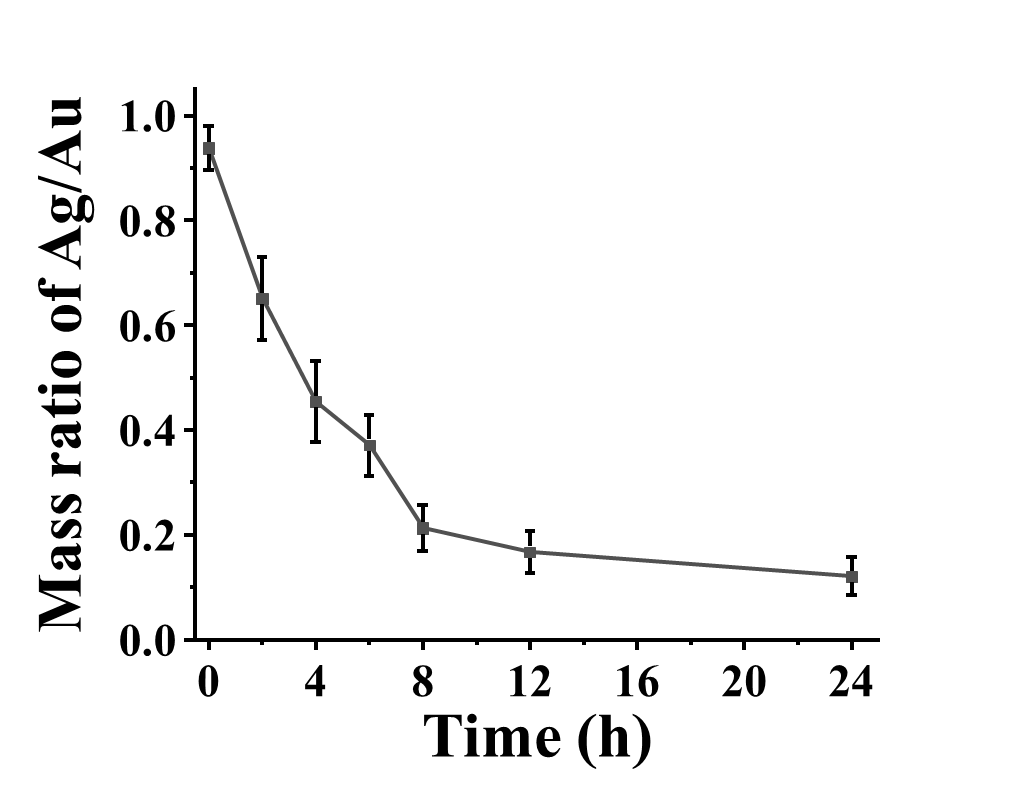


Figure S3. Evolution of the Ag/Au mass ratio throughout the H₂O₂ etching process, as determined by ICP-MS (n = 3).


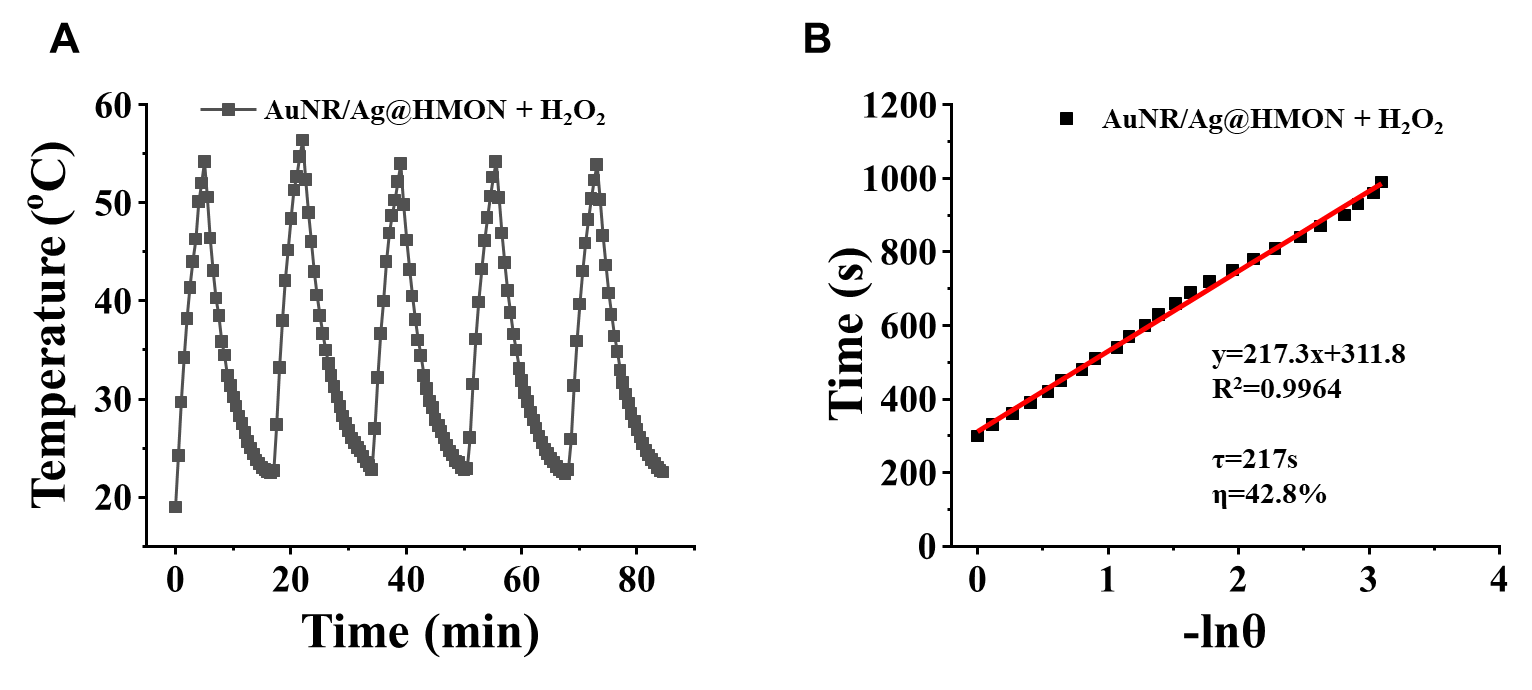


Figure S4. (A) Temperature records of Au/Ag@HMON@CCM etched by H_2_O_2_ over five on/off cycles of 1064 nm irradiation at 0.75 W cm^-2^. (B) The relationship between the linear time data obtained from the cooling time and -lnƟ. Photothermal conversion efficiency is 42.8%.


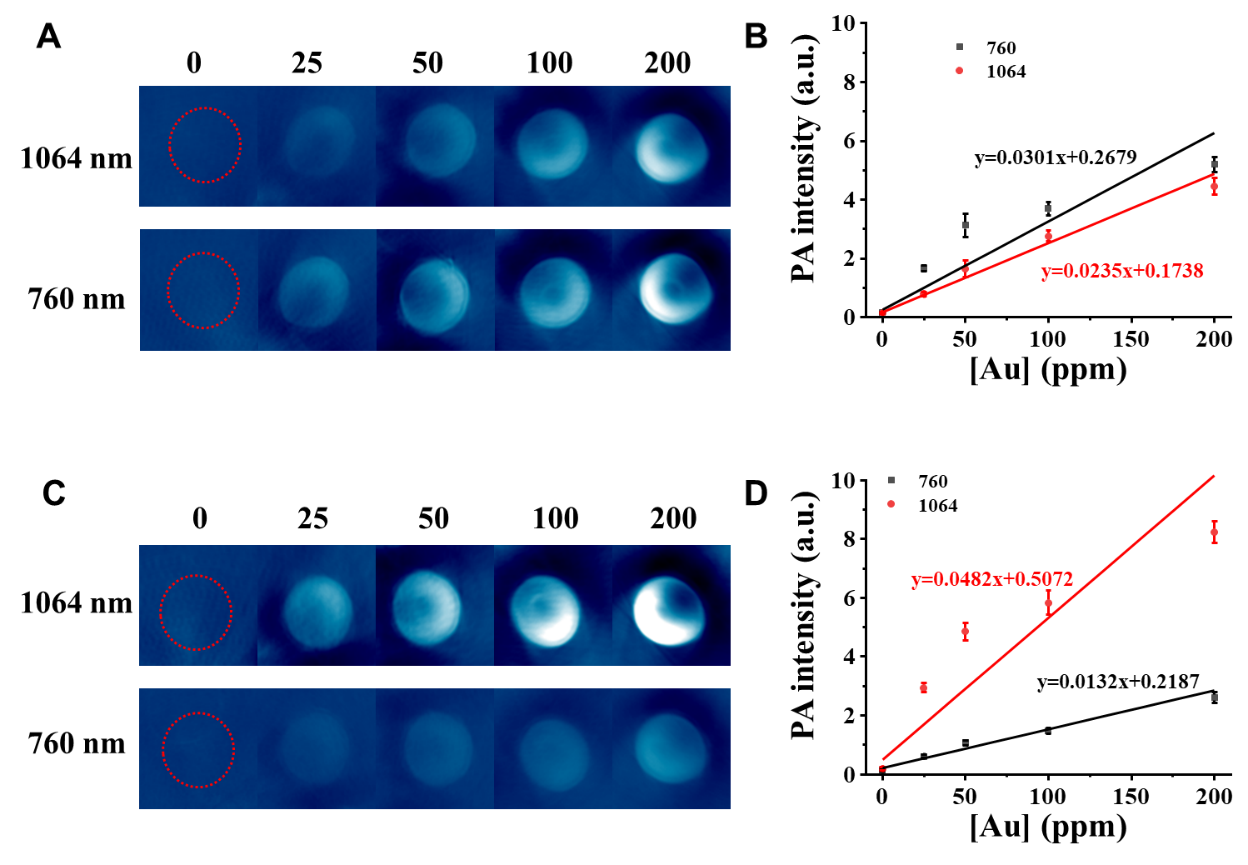


Figure S5. PA images and corresponding PA intensities of phantom filled with Au/Ag@HMON@CCM (A, B) and Au@HMON@CCM (C, D) at different concentrations (0 (saline), 25, 50, 100 and 200 ppm Au) acquired at optical wavelength of 1064 nm and 760 nm (n =3).


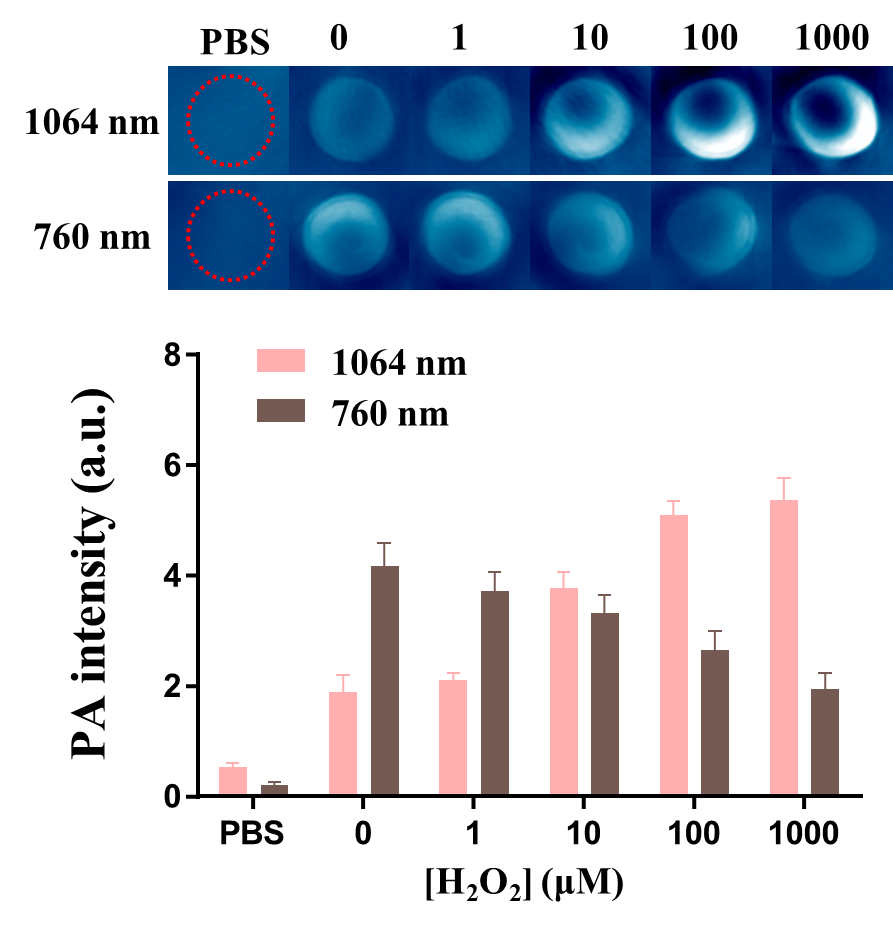


Figure S6. PA images and corresponding PA intensity profiles of the phantom filled with Au/Ag@HMON@CCM ([Au] = 50 ppm) after incubation with different concentrations of H_2_O_2_ (0, 1, 10, 100 and 1000 μM) for 8 h (n = 3).


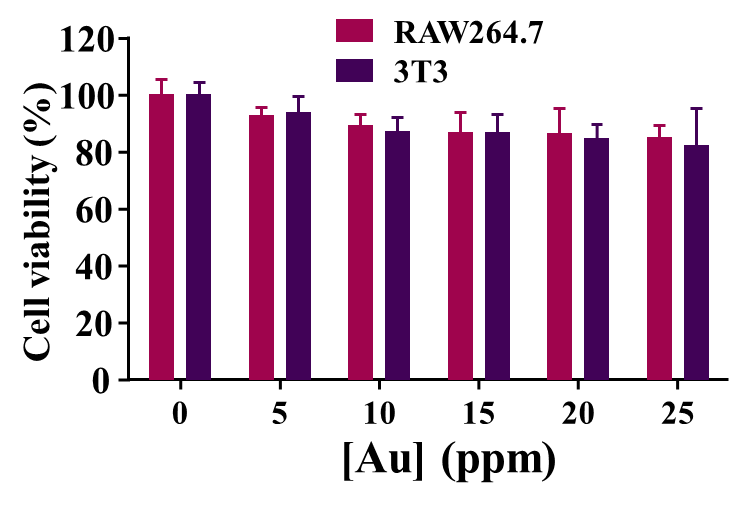


Figure S7. Viability of RAW264.7 and 3T3 cells after incubated with Au/Ag@HMON-NLG@CCM for 24 h (n = 5).


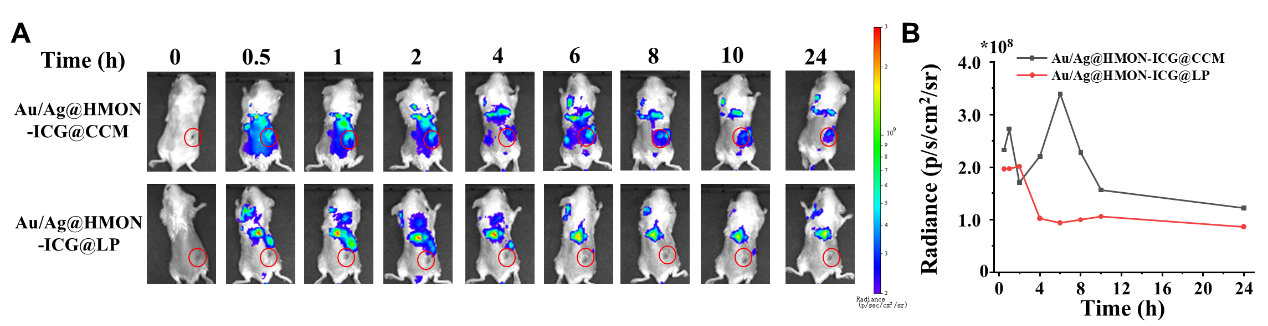


Figure S8. (A) *In vivo* distribution of ICG-labelled Au/Ag@HMON-ICG@CCM and Au/Ag@HMON-ICG@LP at predetermined times (0, 0.5, 1, 2, 4, 6, 8, 10 and 24 h) after intravenous administration via fluorescence imaging and (B) the corresponding fluorescence intensity quantification of tumor sites (n=3).


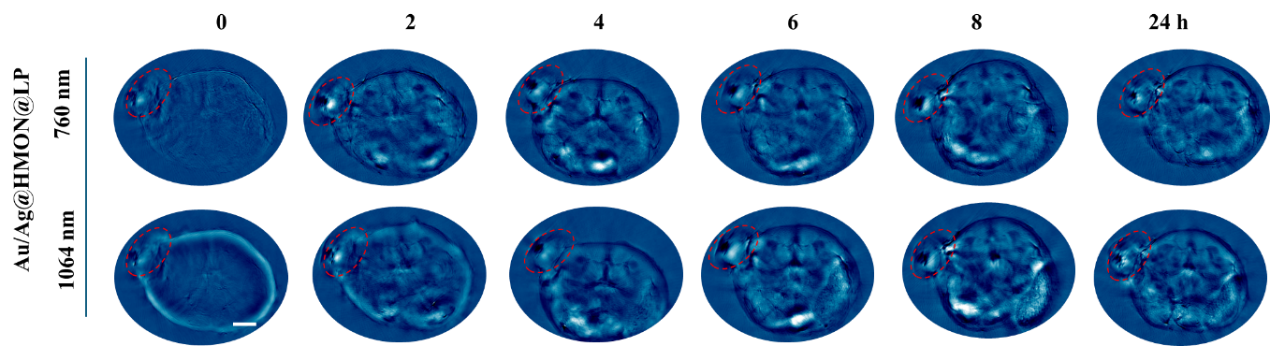


Figure S9. PACT images of Au/Ag@HMON@LP treatment group at different time points post-injection.


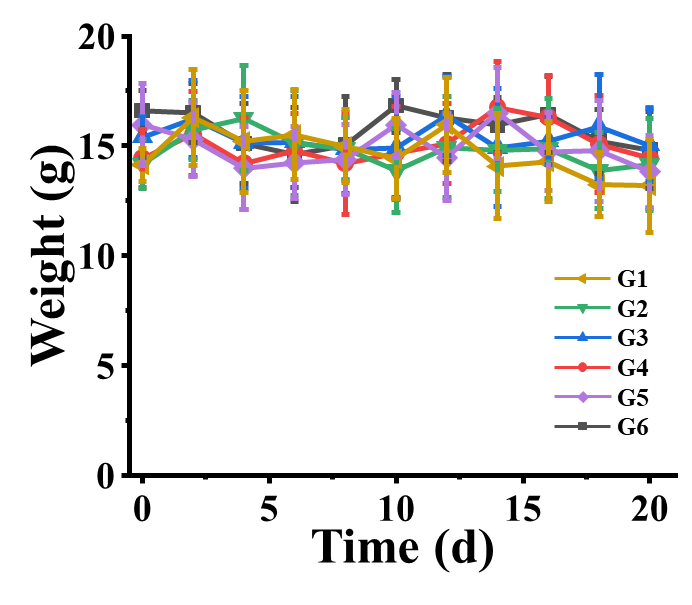


Figure S10. Body weight of mice in different treatment groups (n = 5).


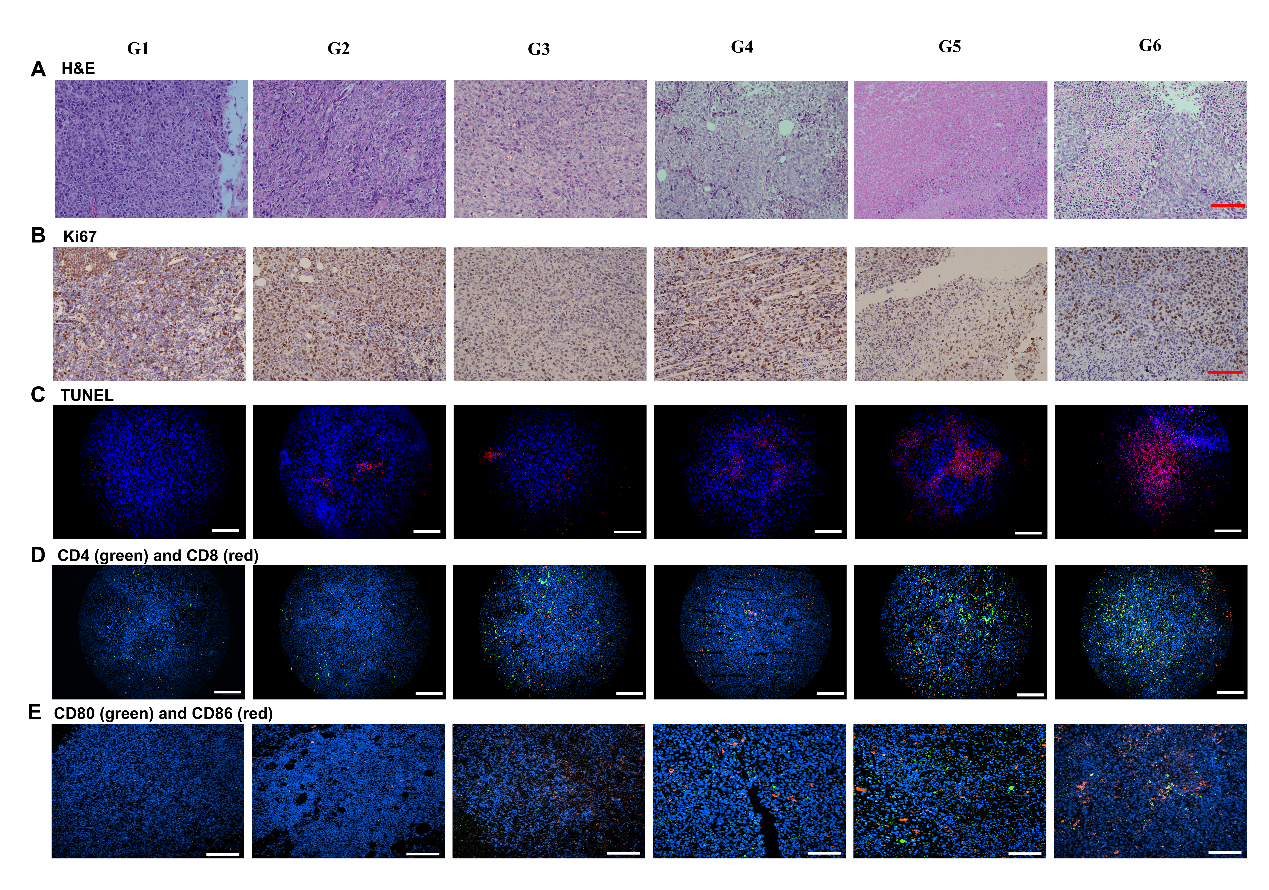


Figure S11. (A) H&E staining, (B) IHC staining via ki67 antibody staining, (C) TUNEL assays staining, (D) CD4 (green), CD8 (red) staining, and (E) CD80 (green), CD86 (red) staining of primary tumor acquired at 21 days from different groups: G1-PBS, G2-Au/Ag@HMON@CCM, G3-Au/Ag@HMON@CCM+Laser, G4-Au/Ag@HMON-NLG@CCM, G5-Au@HMON-NLG@CCM+Laser, G6-Au/Ag@HMON-NLG@CCM+Laser.


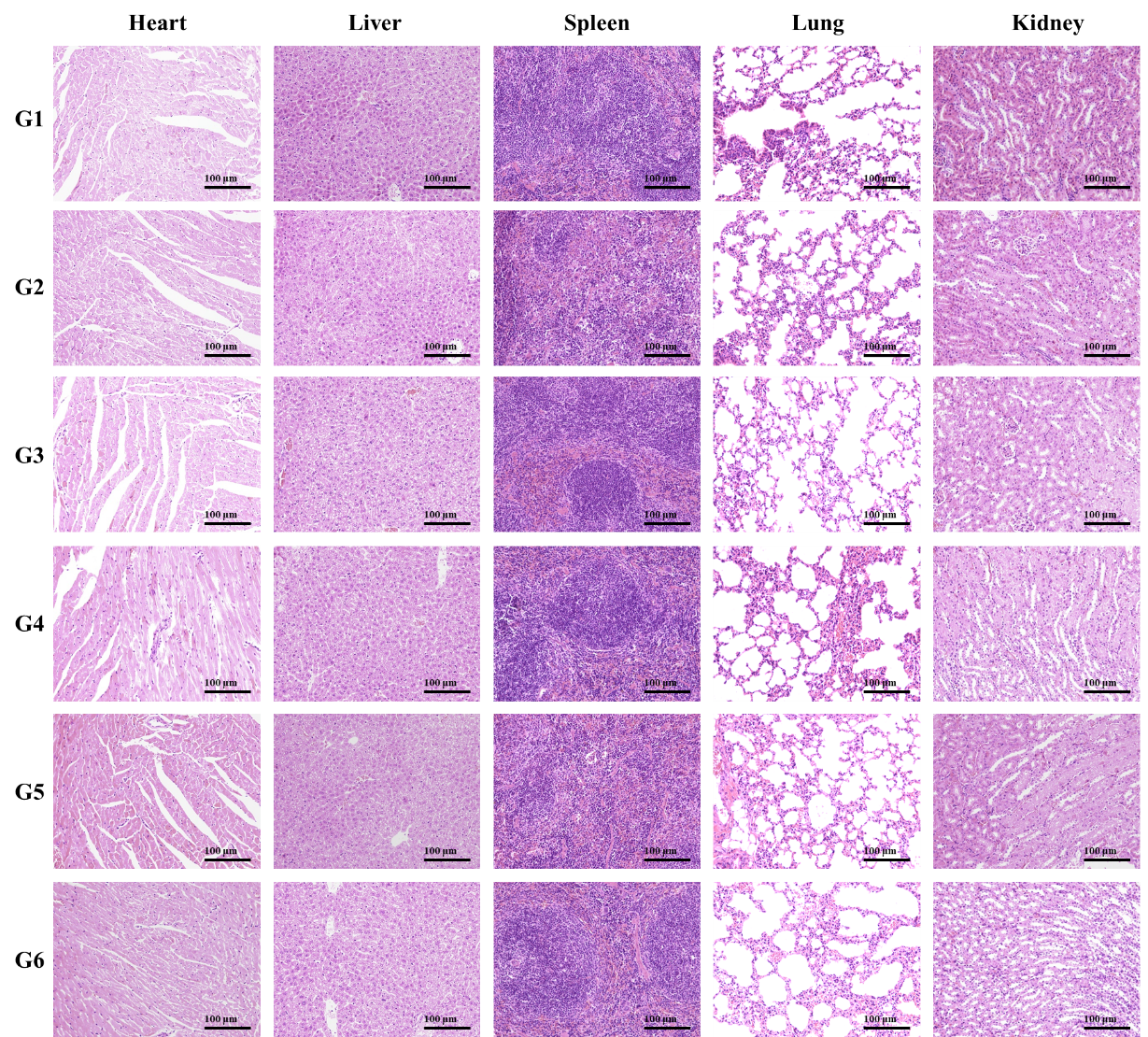


Figure S12. H&E staining images of primary organs sections (heart, lung, liver, spleen, and kidney) from mice post-intravenous administration of various treatments (scale bar: 100 μm).





Figure S13. Survival rate of mice in different groups after injection (n=5).


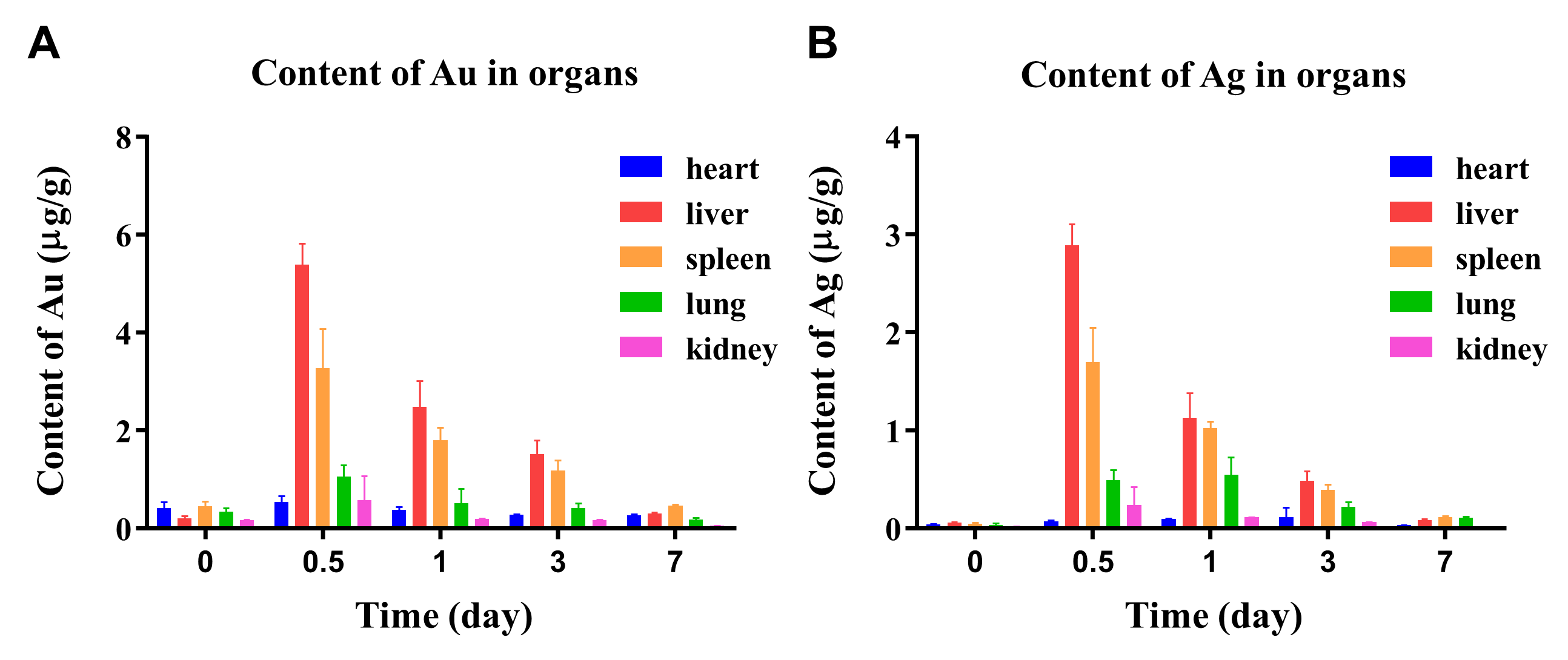


Figure S14. Biodistribution of (A) Au and (B) Ag of major organs of mice prior to injection and at 12 h, 1, 3, and 7 days post-injection (n = 3).


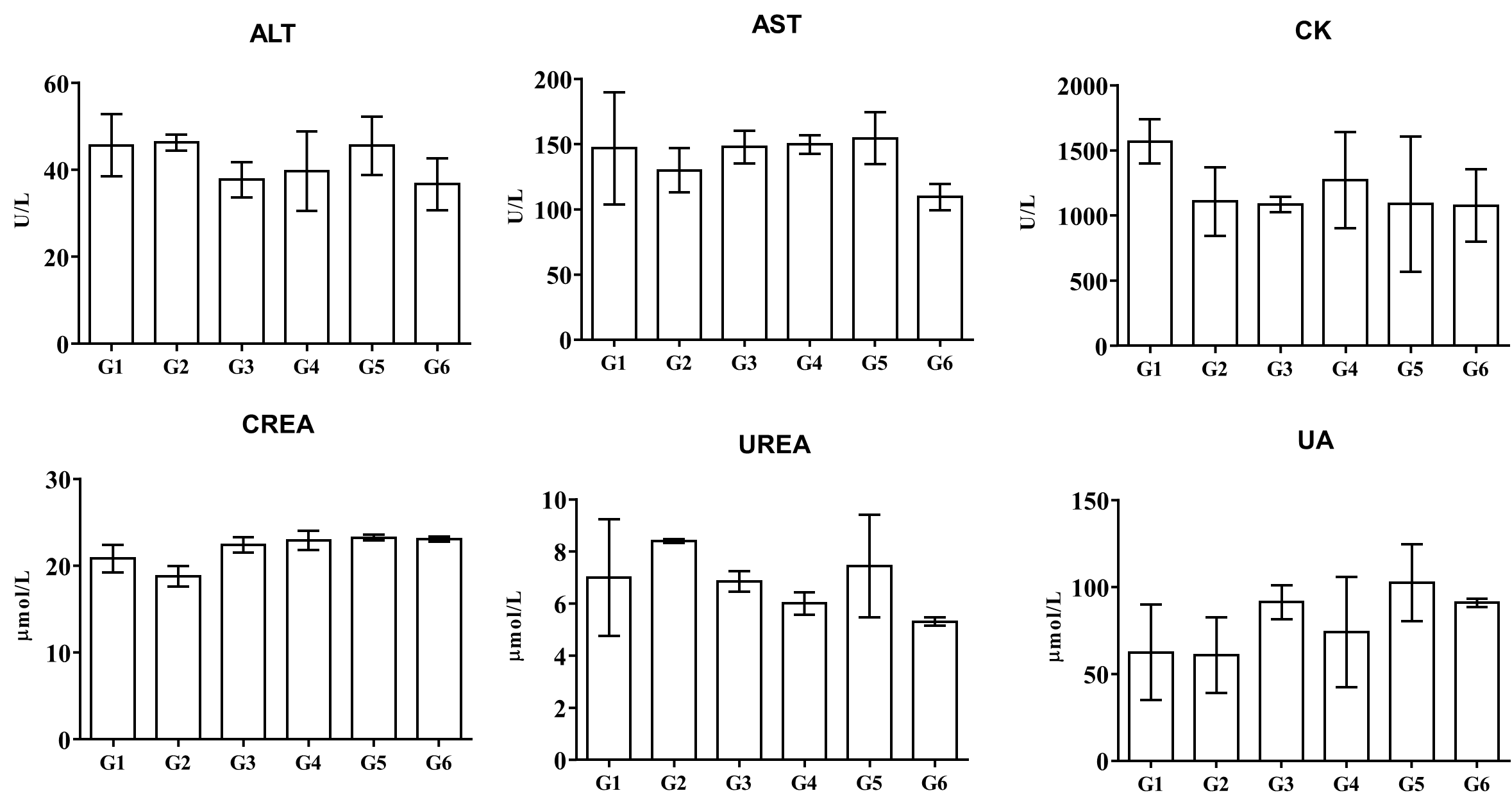


Figure S15. The biochemistry indexes included alanine aminotransferase (ALT), aspartate aminotransferase (AST), creatine kinase (CK), creatinine (CREA), Urea (UREA), plasma uric acid (UA) which were acquired at 21 d after different treatment as indicated (n = 3). Statistical significance was assessed via Student’s t-test (two-tails), ns: no significant difference.


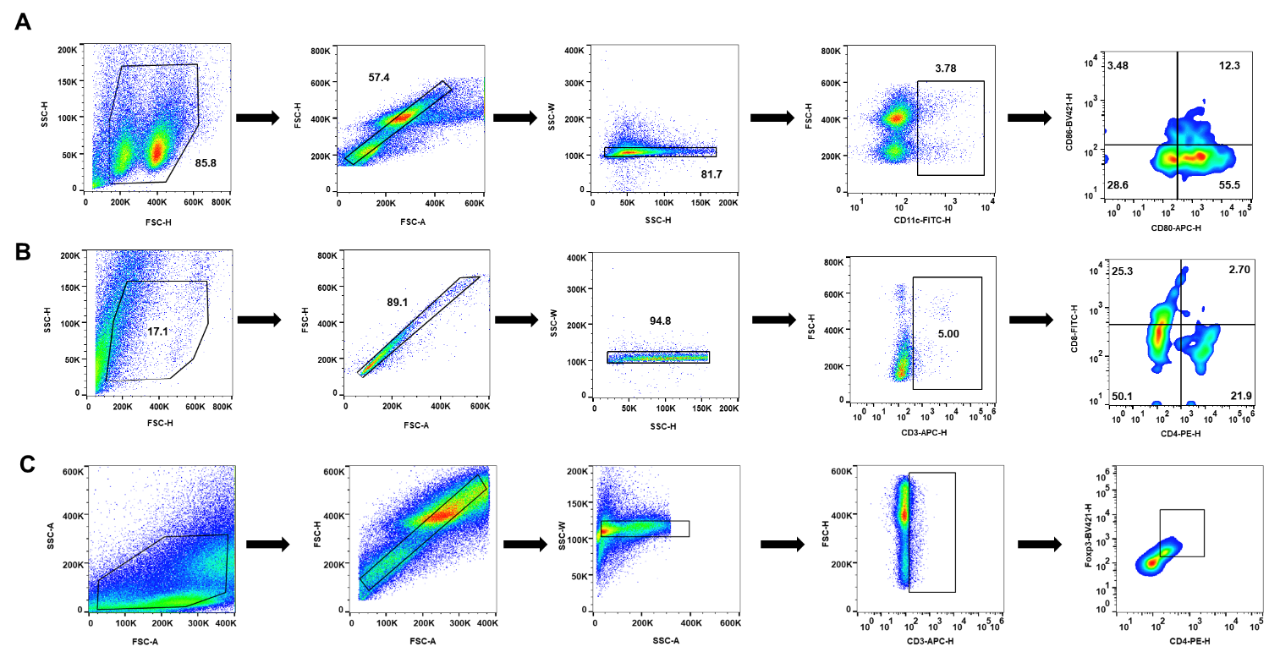


Figure S16. Gating strategies for (A) DCs, (B) T cells and (C) Treg cells subsets.


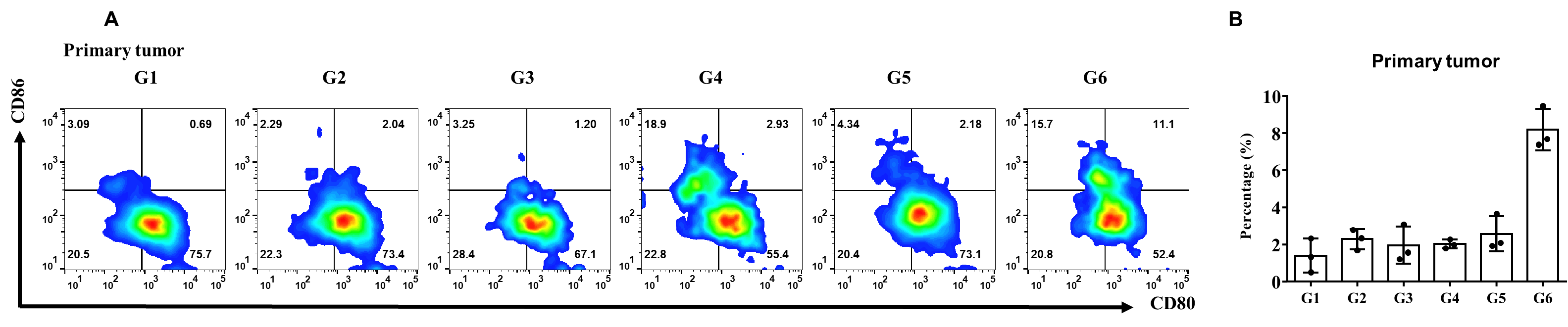


Figure S17. Flow cytometry analysis and quantification of mature DCs (CD80+CD86+) of primary tumors. (Groups: G1-PBS, G2-Au/Ag@HMON@CCM, G3-Au/Ag@HMON@CCM+Laser, G4-Au/Ag@HMON-NLG@CCM, G5-Au@HMON-NLG@CCM+Laser, G6-Au/Ag@HMON-NLG@CCM+Laser).


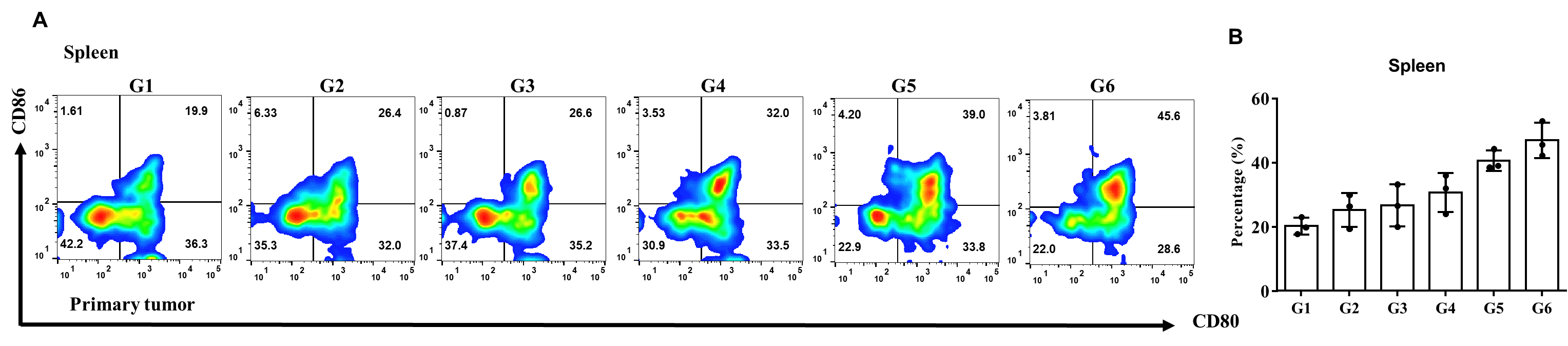


Figure S18. Flow cytometry analysis and quantification of mature DCs (CD80+CD86+) of spleen.
